# Telomeres: Dysfunction, Maintenance, Aging and Cancer

**DOI:** 10.14336/AD.2023.1128

**Published:** 2023-11-29

**Authors:** Pan Liao, Bo Yan, Conglin Wang, Ping Lei

**Affiliations:** ^1^The School of Medicine, Nankai University, Tianjin, China.; ^2^Haihe Laboratory of Cell Ecosystem, Department of Geriatrics, Tianjin Medical University General Hospital, Tianjin, China.; ^3^Tianjin Geriatrics Institute, Tianjin Medical University General Hospital, Tianjin, China.

**Keywords:** telomere dysfunction, TBDs, maintenance mechanisms, age-related disorders, therapeutic, aging hallmarks

## Abstract

Aging has emerged at the forefront of scientific research due to the growing social and economic costs associated with the growing aging global population. The defining features of aging involve a variety of molecular processes and cellular systems, which are interconnected and collaboratively contribute to the aging process. Herein, we analyze how telomere dysfunction potentially amplifies or accelerates the molecular and biochemical mechanisms underpinning each feature of aging and contributes to the emergence of age-associated illnesses, including cancer and neurodegeneration, via the perspective of telomere biology. Furthermore, the recently identified novel mechanistic actions for telomere maintenance offer a fresh viewpoint and approach to the management of telomeres and associated disorders. Telomeres and the defining features of aging are intimately related, which has implications for therapeutic and preventive approaches to slow aging and reduce the prevalence of age-related disorders.

Telomeres are genetic elements found at the terminal positions of linear chromosomes. The TTAGGG repeats that make up telomeric DNA in vertebrates are bound by a group of proteins responsible for controlling their biological activities and preventing them from being recognized as DNA damage. Such recognition can trigger a DNA damage response (DDR), leading to telomere-telomere fusion and genetic instability. The unavailability of telomerase, an RNA-dependent DNA polymerase and reverse transcriptase, renders standard DNA polymerases inefficient in accomplishing complete replication of linear DNA templates. Additionally, due to nucleolytic processing, DNA replication produces chromosomes with progressively shorter telomeres [1]. The phenomenon of telomeres reaching a critical length causes them to lose their ability to bind adequate telomere-capping proteins and become detectable as exposed DNA ends [2]. This event then stimulates DDR pathways, leading to increased expression levels of the cell cycle inhibitors p16 and p21, which in turn restrain proliferation [3,4]. Despite being shortened, these telomeres still maintain an adequate number of telomere-binding proteins to prevent fusion and block DNA repair [5-8]. This process fuels a continuous DNA damage signal, conferring an unalterable arrest in proliferative activities. Eventually, this triggers and preserves cellular senescence, which is a major factor associated with organismal aging and several age-related diseases [9,10]. Initiation of the DDR at telomeres, referred to as tDDR, yields the creation of telomere dysfunction-induced DNA damage foci (TIFs) or telomere-associated DDR foci (TAFs), which are indicators of cellular senescence in both cultured cells and tissues (Table 1). Apoptotic [11,12] or autophagic [13] cell death may occur in some types of cells due to telomere dysfunction.

Cellular senescence is hallmarked by alterations in cellular structures, organelles, gene expression, and chromatin, along with irreversible cell cycle arrest [14]. Notably, the senescence-associated secretory phenotype (SASP), produced by senescent cells, is a complex collection of proinflammatory cytokines. This results in chronic systemic inflammation because it changes the composition of the extracellular matrix, inhibits stem cell activities, encourages cell transdifferentiation, and can propagate the senescence phenotype to neighboring cells [15]. The DDR promotes SASP [16], which can also act in an autocrine and paracrine manner to stimulate the formation of the DDR and TAFs [17-19].

**Table 1:** Comparisons of different TL measurement methods.

Method	Advantages	Limitations	Refs.
**Q-PCR**	easy to conduct; small amount of starting DNA required; many population-based studies for comparisons	large variations among different laboratories, but reproducibility is better in commercial setting; not useful in cancer studies due to aneuploidy; only average TL is provided as a relative ratio	[271]
**TRF (Southern blotting)**	common method for research studies; highly reproducible in some laboratories; many published studies for comparative research	larger amounts of starting DNA required; provides most information on average TL; need to standardize restriction enzymes used to compare studies between laboratories (subtelomeric polymorphisms can alter data obtained); labour intensive	[1]
**interphase Q-FISH**	can be conducted on fixed tissues and cells	labour intensive; TLs expressed as relative fluorescence units (not actually TLs) but using standards measured by TRF actual TLs can be inferred	[272]
**HT Q-FISH**	same as interphase Q-FISH; very reliable and reproducible results; CLIA certified	does not distinguish telomere clustering in interphase cells; does not recognize telomere-free ends	[273]
**Flow FISH**	same as interphase Q-FISH; can provide cell- typespecific information on mostly average telomere lengths; reproducible; CLIA certified	requires an expensive FACS instrument; almost universally uses peripheral blood mononuclear cells (PBMCs)	[274,275]
**metaphase Q-FISH**	can potentially detect telomeres on all chromosomes	does not detect the telomeres that are very short that do not hybridize with probes (appear as telomerefree ends); requires highly skilled cytogeneticist for chromosome-specific analyses	[272]
**STELA**	can detect the shortest telomeres on specific chromosomes	works on only a small subset of individual human chromosomes; low throughput; labour intensive	[276]
**Universal STELA**	measures mainly the shortest telomeres	does not detect larger telomeres and can detect ITSs; manual quantitation; low throughput; labour intensive	[277]
**TeSLA**	measures all the telomeres less than 1 kb and up to 18 kb on all chromosome ends; works on many animal types; automatic quantitation of telomere sizes using user-friendly software	low throughput; labour intensive	[278]

Telomere shortening is insufficient to explicate the senescence of quiescent, nonproliferating, or terminally differentiated cells, despite being conceptually appealing to describe cellular aging and the decline in proliferation. Nonetheless, TAFs and senescence have been observed in postmitotic cells, particularly cardiac myocytes, adipocytes, osteoblasts, neurons, and osteocytes [20-22]. The evolutionary theory that telomere-binding proteins hinder DNA repair in cis [23,24] to preserve the chromosomal linear structure and avoid fusions can be used to elucidate these data. Consequently, telomeric repeat-specific DNA damage (tDD) is resistant to repair, resulting in prolonged tDDR signaling and TAF production at long telomeres [24-26]. The generation of endogenous or exogenous DNA damage is a continuous process in which the portion that arises at telomeres undergoes hardly any repair and therefore accumulates in cells. As a result, this accumulation may trigger a SASP (Fig. 1A).

Furthermore, replicative cellular senescence stimulated by critically short telomeres and the senescence-like conditions induced by broken telomeres in nonreplicating cells share continuous tDDR activation as their common underlying cause (Fig. 1B). Although the mechanisms behind these processes are different, DNA damage at long telomeres could result in the loss or disintegration of the terminal regions of telomeres during the process of organismal aging, thus contributing to the shortening of telomeres.

The premise that DNA represents the sole irreplaceable component of a cell is a compelling argument supporting the central role of maintaining DNA integrity in organismal aging. This is further reinforced by the fact that telomeres are precisely irreparable. Telomeric DNA is difficult to repair and has strong hypersensitivity to oxidative DNA damage; this condition is termed TelOxidation [27]. According to some reports, oxidative stress causes tDD without telomere shortening and quickens telomere shortening in postmitotic (PM) cells [28-30] by suppressing telomerase [31] and blocking the identification of telomere-binding proteins, thereby contributing to the uncapping of telomeres [27,32]. TAF buildup and tDDR activation frequently have a causal relationship with other age-related events. These include epigenetic dysregulation, altered nutrition sensing, repressed autophagy, proteostasis loss, and mitochondrial dysfunction, suggesting a unified "telomere-centric" molecular underpinning for several defining features of aging [33].

**Figure 1.**
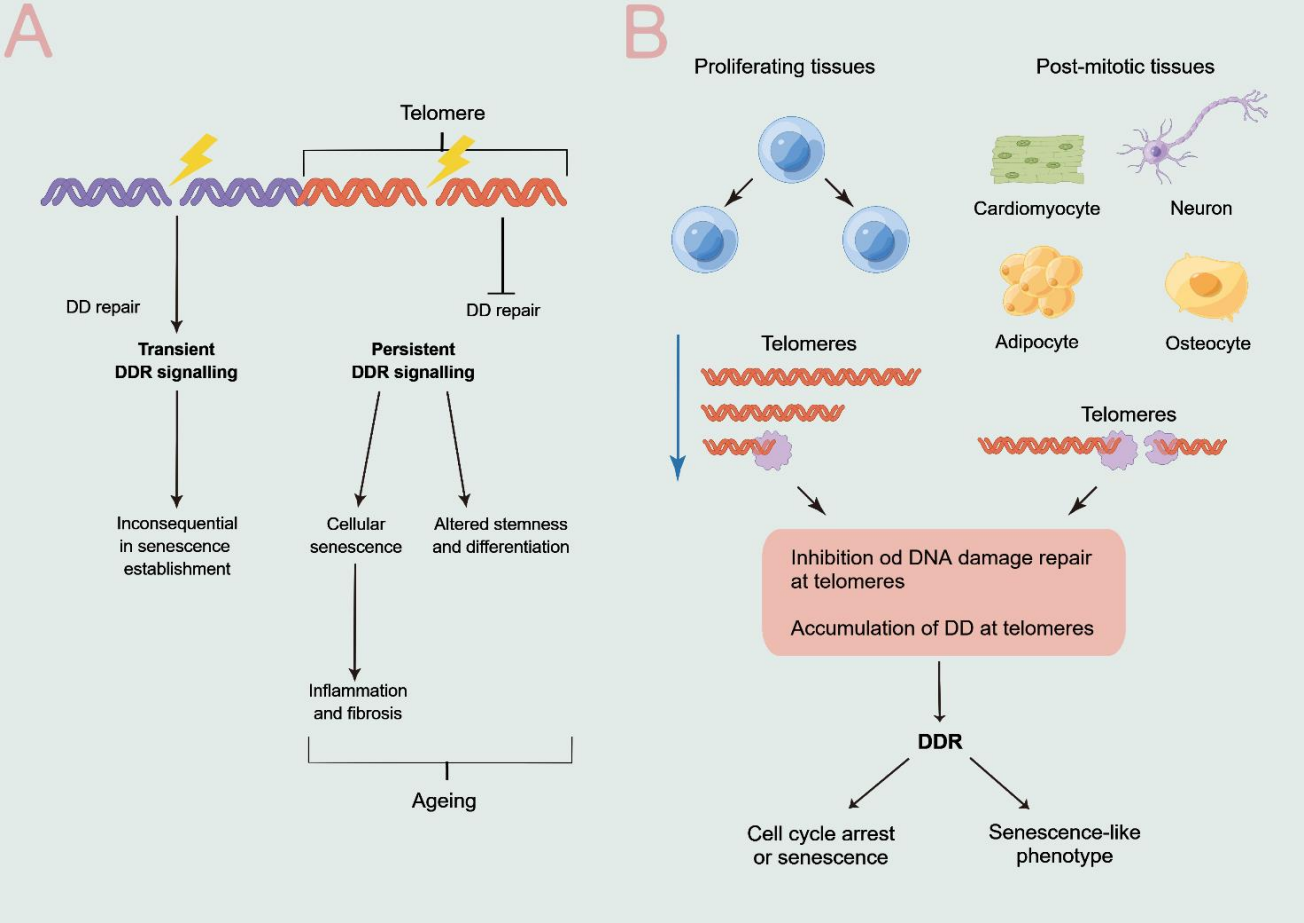
**Telomere damage, shortening, and implications. (A)** A transitory DNA damage response (DDR), which is brought on by genomic DNA damage (DD), might not be enough to produce senescence. Conversely, delayed DDR, along with cellular senescence that is linked to SASP-induced inflammatory response and subsequent fibrosis, results from irreparable, hence permanent, DNA damage at telomeres. The stem cell characteristics are harmed, and differentiation is altered by these processes in a stem cell environment. Altogether, this accelerates the aging of organisms [20]. **(B)** Telomeres in tissues that are prone to proliferation are shortened during cell cycle divisions, and when they get dangerously short, they induce a DDR. Telomere dysfunction in post-mitotic, non-proliferating tissues may be caused by telomeres that have irreversible DNA damage. A senescent phenotype that is defined by halted proliferation and SASP stimulation is maintained in both situations by prolonged DDR activation.

The gradual telomere shortening that occurs as a natural process in humans leads to normal aging and age-associated illnesses [34]. Conversely, a collection of uncommon and diverse Mendelian illnesses [35] known as telomeropathies, telomere biology disorders (TBDs), or short-telomere syndromes are caused by germline anomalies that result either in telomere instability or rapid telomere shortening [36]. As previously discussed, telomeropathy is caused by germline abnormalities in the telomere maintenance genes POT1, RTEL1, PARN, TINF2, DKC, TERC, and TERT [37]. Cellularly, telomeropathy is denoted by (i) lymphocyte immunosenescence, (ii) depletion of hematopoietic stem cells leading to bone marrow dysfunction, and (iii) loss of intestinal stem cells causing intestinal villous atrophy accompanied by intraepithelial lymphocytosis, basal plasmacytosis, villous blunting, and crypt cell apoptosis [38]. At the tissue level, TBDs, particularly Revesz syndrome, Coats plus syndrome, Høyeraal-Hreidarsson syndrome, pulmonary fibrosis, and dyskeratosis congenita (Fig. 2), manifest as a wide spectrum of clinical symptoms, primarily affecting organs and tissues with high proliferative ability and renewal capacity, such as the epithelial and hematopoietic systems. Telomere abnormalities can make cells in some tissues, such as the liver and the lung, which are not known to have high basal proliferative rates more susceptible to environmental stressors, such as a high-fat diet, hypertension, alcohol use, or cigarette smoke [39-41].

**Figure 2.**
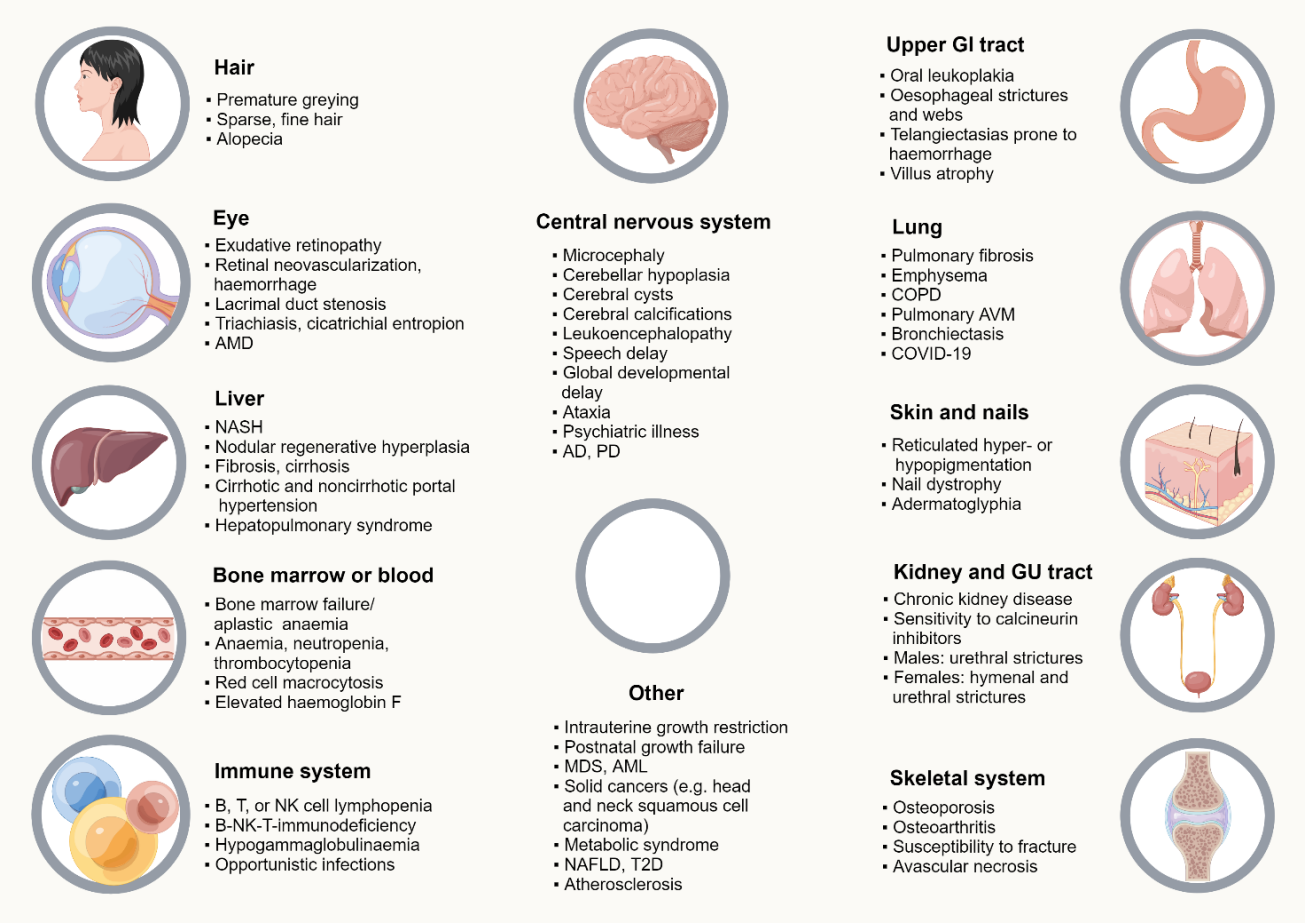
**Summary of clinical features of telomere biology disorders (TBDs)**. Multiple existing reviews and cohort studies have provided detailed descriptions of the clinical characteristics of TBDs [258,258]. Succinctly, the traditional diagnostic criteria for dyskeratosis congenita include nail dystrophy, a triad of mucocutaneous oral leukoplakias, lacy, and coloration of the reticular skin, which most frequently manifest in the second decade of life. Due to gradual bone marrow loss, dyskeratosis congenita is typically identified before these characteristics appear because of improvements in telomere length assessment and genetic screening. An increased loss of hematopoietic stem along with accompanying progenitor cells, which impairs the formation of one or more blood cell lineages, underlies bone marrow failure. Acute myeloid leukemia (AML) or myelodysplastic syndrome (MDS), or other conditions that need hematopoietic stem cell transplant, might develop from bone marrow failure [36,279-281]. Dyskeratosis congenita patients are also at risk for interstitial lung illnesses and solid malignancies (such as carcinomas) [279,282-284]. Other potentially fatal signs include gastrointestinal (GI) hemorrhage, cirrhosis of the liver, and hepatopulmonary syndrome [279,285,286]. Additional characteristics that impact life quality might emerge in several ways (see the Figure for details). In fact, only a few organ systems are exempted, highlighting the significance of telomere biology in a variety of tissues. Three severe TBDs with early onset include Coats plus syndrome, Revesz syndrome, and Hyeraal-Hreidarsson syndrome. The most common symptoms of Hyeraal-Hreidarsson syndrome in patients include fetal growth restriction, acute bone marrow failure that manifests early, cerebellar hypoplasia, and microcephaly [113]. Due to severe infections, immunodeficiency-which is primarily typified by a substantial decrease in B cells and natural killer (NK) cells-is a prevalent cause of mortality during the first few years of life. It is noteworthy that some individuals lack substantial immunodeficiency but have significant neurodevelopmental abnormalities; this distinction is probably genotype-dependent. Bilateral exudative retinopathy (also referred to as Coats disease), postnatal growth failure, GI bleeding, intrauterine growth restriction, osteopenia, bone marrow failure, cerebral calcification and cysts, and scant hair are all characteristics shared by Revesz syndrome and Coats plus syndrome [287-289]. Clinical markers that can differentiate between Revesz syndrome and Coats plus syndrome include the leukodystrophy and more severe bone marrow failure associated with Revesz syndrome, the unique pattern of cerebral cysts and calcifications, extensive gastrointestinal bleeding without a previous history of hematopoietic cell transplantation, and susceptibility to orthopedic fractures in the Coats plus syndrome. They can also be distinguished based on genotypes and how they affect the overall length of telomeres. Last but not least, adult-onset lung disorders like emphysema and pulmonary fibrosis are the most common kind of TBD [282,290,291]. AML, MDS, solid malignancies, liver and renal disorders, GI problems, and bone marrow failure are additional clinical symptoms of adult-onset TBDs. NASH, nonalcoholic steatohepatitis; GU, genitourinary; COPD, chronic obstructive pulmonary disease; AVM, arteriovenous malformation.

**Table 2:** Telomere biology disorder genes, inheritance and primary disease association.

Gene	Main function	Genetic status	Disease associations
**DKC1**	hTR stability	X-linked	Dyskeratosis congenita [45,292-305]
			Høyeraal-Hreidarsson syndrome [306-309]
			Pulmonary fibrosis [301,305,310,311]
			Bone marrow failure or aplastic anaemia [310]
**TERC**	Telomerase RNA scaffold, template	Monoallelic	Dyskeratosis congenita [294,295,312-319]
			Høyeraal-Hreidarsson syndrome [308]
			Pulmonary fibrosis [282,290,294,319-325]
			Liver disease [326-328]
			Aplastic anaemia or myelodysplastic syndrome [294,316,319,327,329-333]
		Biallelic	Pulmonary fibrosis and mucocutaneous features [313]
**TERT**	Catalytic subunit of telomerase, telomere elongation	Monoallelic and biallelic	Dyskeratosis congenita [294,316,334,335,335,336]
		Biallelic	Høyeraal-Hreidarsson syndrome [335-340]
		Monoallelic	Pulmonary fibrosis [282,294,320,321,323,324,341-344]
			Liver disease [326,328]
**NOP10**	hTR stability	Biallelic	Dyskeratosis congenita [345]
		Monoallelic	Pulmonary fibrosis [346]
**NHP2**	hTR stability	Biallelic	Dyskeratosis congenita [347,348]
		Monoallelic	Høyeraal-Hreidarsson syndrome [349]
			Pulmonary fibrosis [350]
**TINF2**	Negative regulator of telomere length	Monoallelic (mostly de novo)	Dyskeratosis congenita [294,351-358]
			Høyeraal-Hreidarsson syndrome [352,359,360]
			Revesz syndrome [308,351,353,361-364]
			Pulmonary fibrosis [365]
			Aplastic anaemia or myelodysplastic syndrome [366-368]
**WRAP53**	Telomerase maturation, activation and trafficking	Biallelic	Dyskeratosis congenita [369-372]
			Høyeraal-Hreidarsson syndrome [373]
			Liver disease [370]
**CTC1**	3′ G-overhang regulation	Biallelic	Dyskeratosis congenita [374,375]
			Coats plus syndrome [285,375-383]
**RTEL1**	Telomere replication, 3′ G-overhang regulation	Monoallelic and biallelic	Dyskeratosis congenita [384-387]
			Høyeraal-Hreidarsson syndrome [384-386,388-394]
		Monoallelic	Pulmonary fibrosis [321,325,341,344,395-398]
		Monoallelic and biallelic	Aplastic anaemia or myelodysplastic syndrome [387,392,399]
**ZCCHC8**	hTR maturation/stability	Monoallelic	Pulmonary fibrosis [400]
			Bone marrow failure [400]
PARN	hTR maturation/stability, stability of telomere-factor transcripts	Biallelic	Dyskeratosis congenita [401,402]
			Høyeraal-Hreidarsson syndrome [349,403-406]
		Monoallelic and biallelic	Pulmonary fibrosis [319,321,325,341,395,403,407,408][344,409-411]
		Monoallelic	Kidney disease [412]
**ACD**	Telomerase recruitment via TEL patch and NOB domains, telomerase activity/RAP	Biallelic	Høyeraal-Hreidarsson syndrome [413,414]
		Monoallelic and biallelic	Bone marrow failure or aplastic anaemia [258,258]
		Monoallelic	Pulmonary fibrosis [415,416]
**POT1**	3′ G-overhang regulation, telomerase activity/RAP, telomere stability	Biallelic	Coats plus syndrome [417]
		Monoallelic	Pulmonary fibrosis [418]
**NAF1**	hTR stability	Monoallelic	Pulmonary fibrosis [419]
			Myelodysplastic syndrome [419]
			Liver disease [419]
**STN1**	3′ G-overhang fill-in	Biallelic	Coats plus syndrome [285,420-422]
**RPA1**	Telomere replication?; regulation of telomerase activity?	Monoallelic	Pulmonary fibrosis [423]
			Dyskeratosis congenita [423]
			Myelodysplastic syndrome [423]
			Immunodeficiency [423]
**DCLRE1B**	3′ G-overhang regulation, telomere stability	Biallelic	Dyskeratosis congenita [424]
			Høyeraal-Hreidarsson syndrome [424]

Only patients for whom molecular sequencing was performed and reported are included. hTR, human telomerase RNA; RAP, repeat addition processivity.

The components and mechanisms implicated in telomere preservation, morphology, and function have been extensively studied using a variety of mouse models [42]. Nonetheless, the extrapolation to humans of data captured in murine models may be constrained by species-specific characteristics. Mice, for instance, have short life expectancies (approximately 2-3 years), residual TERT expression in multiple adult tissues [43], and long telomeres (approximately 30-150 kb) in typical Mus musculus laboratory strains [44]. Hence, the identification and analysis of the genetic factors responsible for human TBDs are critical in enabling progress regarding our comprehension of the biological mechanisms of telomeres in the context of human health and disease by identifying and analyzing the genetic origins of TBDs in humans. In 1998, it was discovered that the genetic etiology of dyskeratosis congenita, a TBD, was a detrimental mutation in the DKC1 gene [45]. Sixteen additional TBD-causing genes have since been identified (Table 2). As expected, they all have an impact on telomere biology, but they do so by affecting different facets of telomere structure, preservation, and functionality.

## TELOMERE MAINTENANCE CONTROL

### Shelterin and the telomerase complex

**Figure 3.**
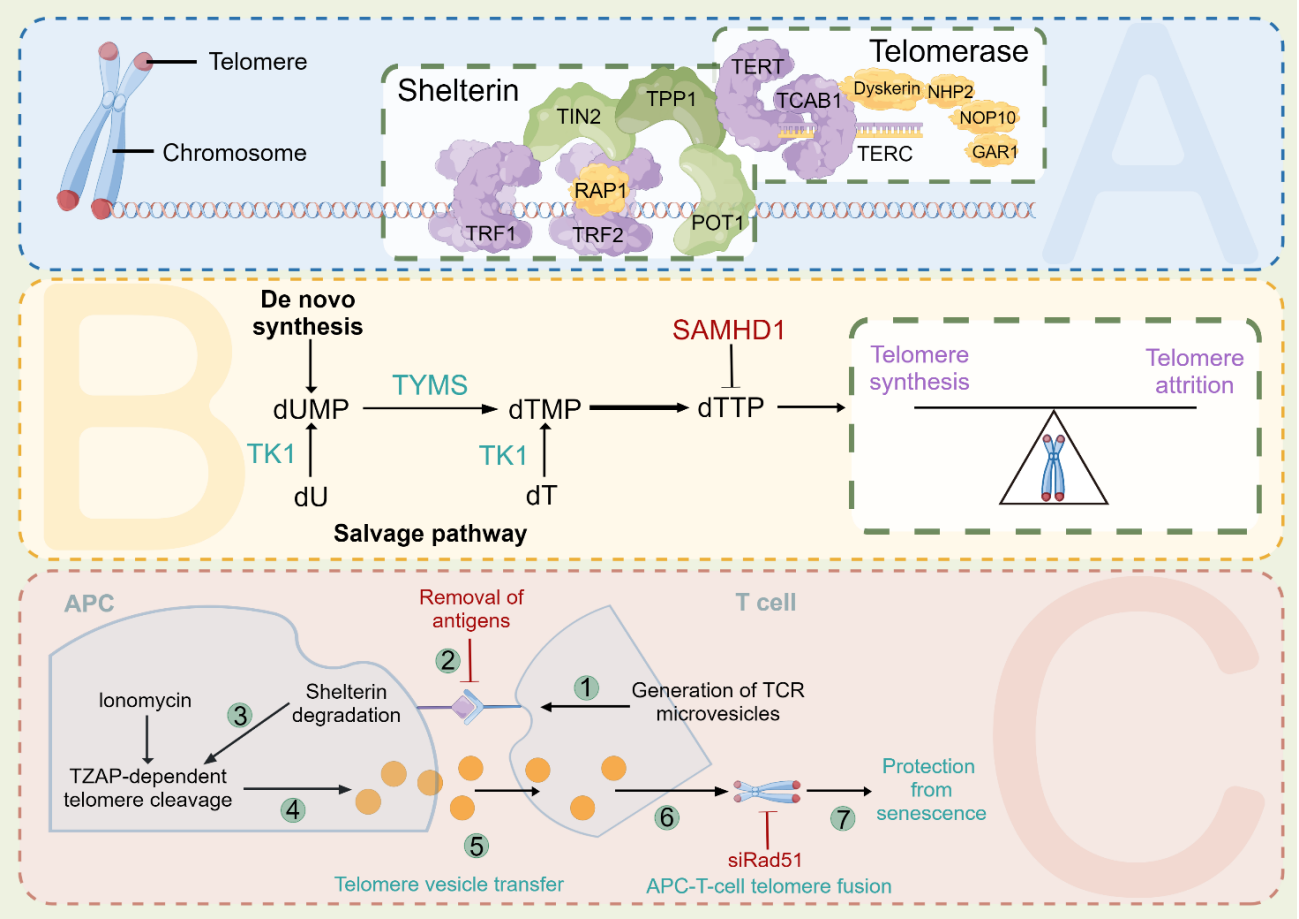
**Controls of telomere maintenance. (A)** Sheltertin and the telomerase complex. **(B)** The relationship between thymidine nucleotide metabolism and telomere length. **(C)** Telomere vesicle transfer.

Chromosome integrity, which is crucial for maintaining species survival and reproduction, is maintained by telomeres. The functions of telomere end protections have been conserved throughout evolution and extend from lower multicellular organisms such as T. thermophila to higher-order organisms such as Homo sapiens [46,47]. In terms of structure, telomeres are composed of tandem repeat sequences of TTAGGG, which may range in length from several kilobases to tens of kilobases. These sequences lead to the termination of the telomeres at the 3' end, leaving behind a single-stranded overhang consisting of 75-300 nucleotides that are particularly rich in guanine nucleotides (Fig. 3A). Griffith and de Lange's groundbreaking research demonstrated that the overhang bends backward on itself to produce a lariat-like structure known as the T loop [48].

A specific group of proteins that is commonly referred to as the shelterin complex encapsulates the telomeres. This complex comprises six subunits of proteins, including RAP1, TIN2, POT1, TPP1, TRF2, and TRF1, that interact to form a multimer [49]. This higher-order telomere morphology controls telomerase entry and functions at the termini to prevent DNA repair processes from fusing ends, mostly through recombination or conventional/alternative nonhomologous end joining, and suppresses DNA damage signaling from the telomere ends. Alterations to the components mentioned above may impair the shelterin-telomere complex, leading to end fusions as well as early senescence [50]. Similarly, excessive TRF1 expression or POT1 deregulation disrupts the ability of telomerase to bind telomere terminals, thus leading to a reduction in telomere length [51,52]. In contrast, owing to the inability of TRF1 to recruit the Bloom (BLM) helicase and thus prevent BLM from performing vigorous double-strand break repair during replication, a loss of TRF1 expression also causes the development of common fragile sites in telomeric DNA [53].

Germ cells [54] together with undifferentiated progenitor and stem cells of the skin [54,55], intestinal tract [56], hematopoietic system [57], hair follicle bulge [58], and testes [55] have elevated levels of telomerase expression compared to normal tissues. TERT levels are low or undetectable in differentiated cells, such as spermatids, fibroblasts, cardiac myocytes, neurons, skeletal myocytes, and keratinocytes [59,60].

TERC is associated with small nucleolar RNAs (snoRNAs) and small Cajal body RNAs (scaRNAs). Although these RNAs are encoded in the introns of other genes, the prototype gene TERC encodes its promoter [61]. The precursor format of TERC RNA transcription includes a poly (A) tail and a 5' methylguanosine cap. The disease-associated poly (A)-specific ribonuclease (PARN) deadenylates these precursors, which subsequently stimulates TERC to mature and aggregate [46,47]. A three-nucleotide sequence widely recognized as the CAB box, found in the H/ACA domain of TERC, is necessary for the protein to bind to telomerase Cajal body protein 1 (TCAB1). The catalytic activity of telomerase and its movement to Cajal bodies, which facilitates its movement to telomere ends, depends on TCAB1. Telomerase interacts with TERC via its CR4/5 and PK/T motifs. Dyskerin, NHP2, NOP10, and GAR1 are only a few of the proteins necessary for the holoenzyme to function properly. These proteins, together with other proteins, form its key components.

Collectively, telomere morphology, along with tightly controlled telomerase activity and recruitment, guarantees optimal telomere preservation in normal cells. Each of these characteristics is also susceptible to mutations and dysregulation, which can result in sporadic and familial disorders. However, there are still many unanswered concerns about the mechanisms through which the telomerase complex can detect and be recruited to the shortest telomeres, as well as the precise sequence involved in the successive assembly of various components. Furthermore, it is unclear how these mechanistic actions could be modulated differently in healthy and developing neoplastic cells. To characterize the involvement of telomerase in the normal aging process as well as in hereditary and somatic degenerative disorder etiology in humans, a more in-depth comprehension of the control of telomerase expression and activity is crucial.

### Metabolism of thymidine nucleotides

Impaired telomere length maintenance is associated with shortened lifespan [62] and fatal genetic degenerative diseases [63-66]. The metabolism of thymidine nucleotides was discovered to be a crucial process regulating the homeostasis of human telomere length by performing genome-wide CRISPR-Cas9 functional telomere length screening in intact cells (Fig. 3B) [67]. The balance of catabolism, salvage, and de novo synthesis tightly regulates the levels of DNA precursors [68-70]. Telomere length is extremely susceptible to thymidine nucleotide metabolic alterations: the deletion of the dNTP-degrading gene SAMHD1 prolonged telomeres, but the deletion of genes related to thymidine nucleotide production or the salvage pathways decreased telomere length [67]. Telomere synthesis is extremely sensitive to small molecules that interfere with the metabolism of thymidine nucleotides as well as genetic changes. Strong telomere extension was induced in cells when dT was added; however, telomere repeat synthesis by telomerase was inhibited by 5-fluorouracil (5FU) or hydroxyurea treatment, which restricted the formation of dTTP [67]. Taken together, these studies emphasize the crucial significance of thymidine nucleotide metabolism in maintaining optimal telomere length in humans, which aligns with recent data from population studies and Mendelian genetic evidence [62,71,72].

Although dTTP, dATP, and dGTP are canonical substrates of human telomeres, the significant effect of thymidine nucleotide metabolism on telomere length in human cells is unanticipated, as previous research on reconstituted telomerase [73-76] and yeast [77,78] has shown that dGTP is the limiting factor for telomerase activity. Recent evidence for substrate-independent augmentation of telomerase activity by dT nucleotides was discovered utilizing a modified telomerase enzyme that does not use dTTP as a substrate in vitro or in live cells [67]. Although secondary effects of dT on dATP and dGTP levels cannot be completely ruled out as a contributing factor to changes in the telomere length of cells, a substrate-independent effect of dTTP on telomerase activity that may be allosteric in nature provides a comprehensive explanation for these biochemical, pharmacological, and genetic observations [62,71,72]. Moreover, this effect, along with recent human genetic data, firmly confirms the role of the thymidine nucleotide metabolic process in controlling telomere length. Particularly, a preponderance of the relevant orthogonal evidence, incorporating genome-wide association studies (GWASs), 5-FU therapy, high-throughput functional genetic screening, and genetic research, jointly connotes the thymidylate synthase gene (TYMS) as a crucial control point, hence demonstrating a restrictive function for de novo dT nucleotide synthesis in the control of telomere length in humans.

Replication stress signaling has previously been linked to higher recruitment of telomerase to telomeres [79] and lengthened telomeres [80,81]. The data indicate that the lengthening of telomeres via dT may occur at optimum concentrations without causing replication stress or cell cycle arrest. Additionally, the compounds 5-FU and hydroxyurea, which are recognized to generate replication stress, were shown to prevent telomere repeat formation by telomerase in the system instead of lengthening telomeres [67]. It is possible that changes in telomerase recruitment due to replication stress may contribute to telomere extension at very high dT dosages; nonetheless, evidence demonstrates that altering dT nucleotides at more physiologically relevant concentrations may affect the length of human telomeres in other ways through other mechanisms, including the activation of telomerase [67].

Cell cycle suppression and replication stress have been documented as insufficient explanations for telomere elongation from dT. High dosages of dT, which are frequently used to synchronize cells, were discovered to produce significant alterations in the production of telomere repeats [67]. To simplify telomerase biology measurements, dT or associated compounds, such as bromodeoxyuridine, have been used in several studies of the biology of human telomerase to measure the timing of telomere production and repeat addition at chromosomal terminals during the cell cycle. The findings suggest that a more profound understanding of telomere biology impacts may be necessary for the use of dT as well as its analogs for synchronization or labeling in human cells [67].

Diseases such as cancer and mitochondrial genetic abnormalities have been linked to defective synthesis of nucleotides [68,82,83], and numerous life-saving treatments, such as those for cancer, autoimmune and viral disorders, have been associated with nucleotide metabolic modification [84,85]. Notably, dT supplementation encourages telomere extension quickly at low micromolar concentrations. This impact has been shown in a wide range of cell lines, including induced pluripotent stem cells (iPSCs) obtained from patients with TBDs brought on by varied, hypomorphic genetic abnormalities. The results obtained from in vitro experiments and studies conducted on cultured cells, in addition to those of ongoing clinical trials that evaluate the efficacy of oral dT supplementation in treating mitochondrial genetic disorders [86], have suggested the possibility of a therapeutic opportunity to modulate telomere length by regulating thymidine metabolism in individuals affected by various genetic degenerative disorders [67].

DNA replication and repair are frequently viewed in connection to dNTP metabolism. The activity of telomerase reverse transcriptase is particularly sensitive to the homeostasis of thymidine nucleotides [67]. Although DNA polymerases [87] and human immunodeficiency virus type 1 reverse transcriptase [88] may both be inhibited by dT supplementation monotherapy, dT therapy caused telomere formation to increase significantly by telomerase [67]. These discoveries contribute to our growing understanding of how variations in the shared pool of cellular nucleotide substrates, which are determined by genetic diversity and other factors, may have a variety of impacts on the diverse DNA synthesis mechanisms in cells. Maintaining telomere length, nuclear and mitochondrial genome integrity, and other influences, such as the limitations of endogenous or exogenous retroelements, have probably been trade-offs in evolutionary constraints on dNTP metabolism. A fresh perspective on the genetic modulation and development of DNA precursor metabolic activities in humans is provided by telomere length homeostasis.

### Transfer of intercellular vesicles

Uncertainty surrounds the formation of senescent T-cells. A theoretical framework has been proposed in which telomeres are transferred from antigen-presenting cells (APCs) to T-cells as a means of protecting these recipient T-cells from undergoing replicative senescence (Fig. 3C) [89]. A naïve or central memory T-cell is the preferred recipient. Upon acquiring telomeres from APCs during antigen presentation, recipient T-cells undergo a transition toward a stem-like/central long-lived memory status. Conversely, T-cells that fail to acquire telomeres become skewed toward replicative senescence instead [89].

While the proposed theoretical framework suggests that the fate of certain T-cells in terms of aging is established even before cell division commences, other pathways are available for the generation of memory T-cells. For example, memory T-cells can be produced in a sequential manner through the contraction of an immediate effector response and a corresponding metabolic shift from glycolysis to oxidative phosphorylation [90]. Alternately, asymmetric division could be used to produce the memory cell straight from the naïve cell, circumventing an effector stage [91]. Additionally, it is conceivable that distinct T-cell populations exhibiting stem-like characteristics are the source of all memory cell types [92].

It is unclear how APC-telomere-containing T-cells divide in response to telomere transfer; nevertheless, telomere transfer may affect how these T-cells divide and differentiate following antigen activation, leading them to differentiate/divide linearly [93] and/or asymmetrically [91]. The quantity of telomere transfer and eventual T-cell differentiation may be influenced by antigen potency. Despite identical antigen specificity, a significant proportion of T-cells fail to acquire telomeres from APCs and are directed toward a short-lived effector state, which may contribute to the development of senescent progenitors. Thus, mechanisms beyond T-cell receptor specificity must be in place to regulate telomere transfer in response to antigen presentation.

It has been speculated that T-cells undergo senescence following multiple antigen-stimulating episodes [94] when telomerase is no longer activated by highly differentiated effectors and proliferation stops. T-cells without telomeres may indeed multiply, albeit to a lesser level than T-cells with telomeres, and thus, a linear senescence mechanism is plausible. Conversely, a different hypothesis for the development of senescent T-cells proposes that T-cells are predisposed to senescence after just one unsuccessful attempt to transfer telomeres in the process of antigen stimulation [89]. T-cells are supported by telomerase, and herein, the telomere transfer mechanism at two distinct stages of the activation of T-cells is disclosed. In an antigen-specific mechanism, telomere transfer occurs when T-cells are still bound to APCs and establish immune synapses. Telomerase subsequently acts once the synapse breaks, causing the T-cells to experience tremendous proliferation [89]. Hence, telomere transfer increases specific, probably ultrashort, telomeres by ~3,000 bp before the commencement of cell division, whereas telomerase replaces telomere depletion at all chromosomes after postsynaptic T-cell division (by approximately 100-200 bp) [89].

In summary, findings show that critical decisions about the aging fate of T-cells are determined as soon as they make their first synaptic connections with APCs, awaiting telomere transfer. As previously proposed, T-cell senescence and final differentiation must be caused by as-of-yet unidentified signals [95]. the signal is currently understood to be telomere transfer [89]. This is distinct from the notion that senescent T-cells accumulate after long-term viral infections, aging, and malignancy [94,96,97] since these cells lack the capacity for antigen-specific proliferation in contrast to T-cells containing APC telomeres. There is some debate about whether senescent T-cells are likely to be resistant [98] or susceptible to the apoptotic process [99] and what fundamental process is causing their production. Senescent T-cells, or their progenitors, might be transient cells produced continuously by activation events without telomere transfer. Consequently, the immunological synapse plays a crucial role in determining the immediate destiny of T-cells in terms of senescence, although this function has not yet been precisely defined [89]. This theoretical framework acknowledges the possibility that memory cells might also originate from effector cells through a dedifferentiation process [100], which has been demonstrated to confer stem-like characteristics [100] to the resulting dedifferentiated cells, similar to those T-cells that acquire telomeres from APCs.

The proposed process of intercellular transfer of telomeres represents an alternative mode of decentralized immunity [101], wherein APCs redistribute telomeres in a way that encourages certain T-cells to develop into long-lasting memory cells, thereby avoiding senescence. The concept of decentralization suggests that T-cells do not rely solely on the telomerase enzyme for telomere extension. It remains to be seen whether memory T-cells, which are produced without the involvement of telomere transfer, exhibit similar long-term survival capacity as their telomere-acquiring counterparts.

## CROSSTALK OF INDICATORS OF CELLULAR AGING WITH DYSFUNCTIONAL TELOMERES

**Figure 4.**
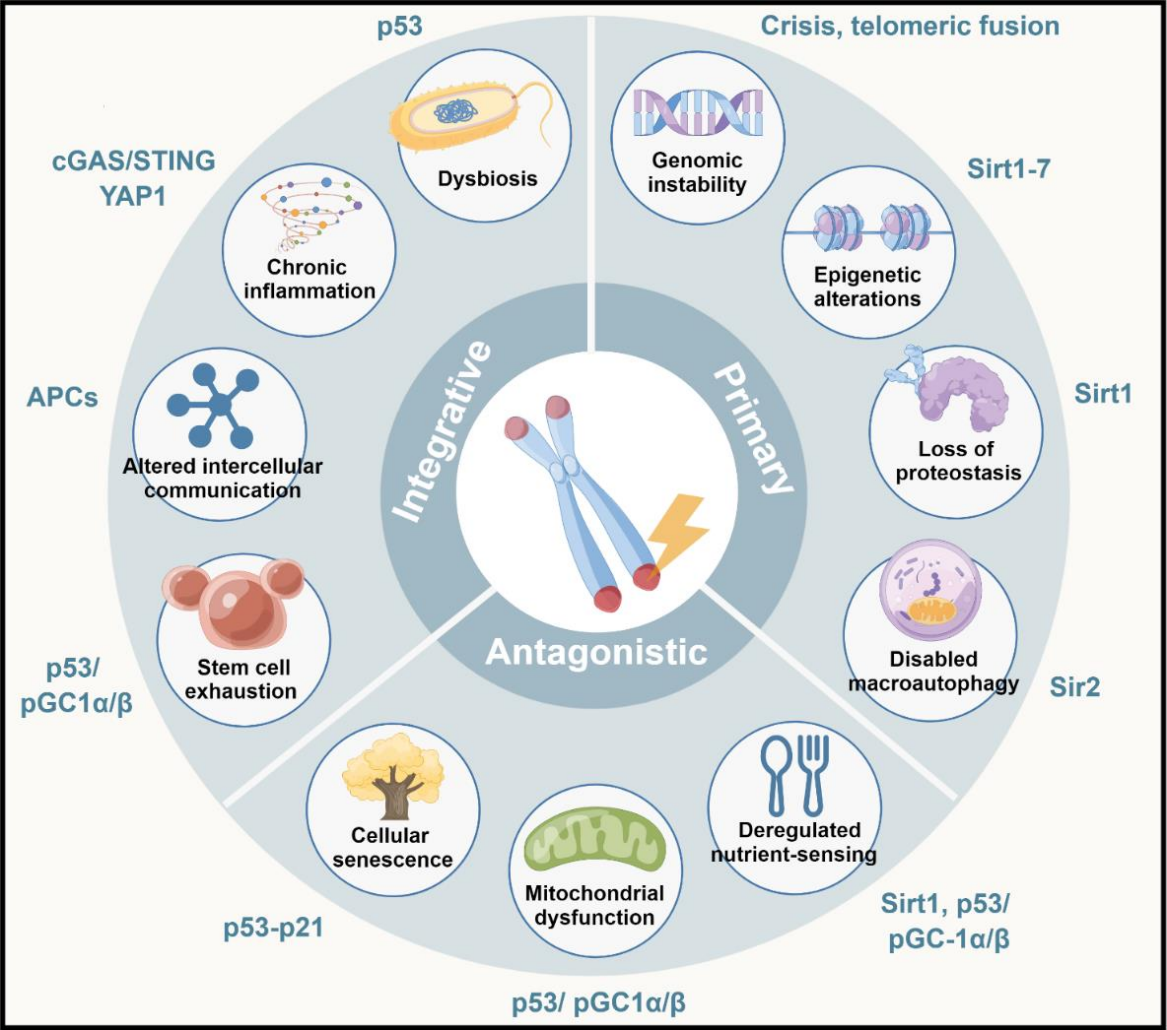
Relevance of telomere dysfunction to cellular aging hallmarks.

Limiting telomere reserves acts as a barrier to cellular immortalization, although functional degradation of telomeres is accompanied by both aging-related fitness reduction and cancer-causing genomic instability. Notably, one of the twelve molecular and cellular indicators of senescence has been cited as telomere disruption [102,103]. In this section, we probe the function of telomeres in the aging process in depth with a focus on how these structures interact with the other signs of aging to either fuel or amplify these mechanisms (Fig. 4). We also provide a summary of how genetic model systems have helped us understand how telomeres interact with various aging underpinnings and pathways, including those causing premature aging disorders. The connections between telomere dysfunction and the underlying mechanisms of each hallmark of aging are discussed in the subsections below.

### Dysbiosis

The synthesis of vital metabolites such as vitamins, amino acid derivative products, secondary bile acids, and short-chain fatty acids (SCFAs), as well as nutrient digestive processes, pathogen defense, and immune function, all depend on the gut microbiome, which has recently been identified as a key player in many physiological functions. Additionally, the peripheral and central nervous systems, as well as other distal organs, receive signals from the gut microbiota, which has a significant influence on the general regulation of host health [104]. Dysbiosis is caused by the impairment of this bacteria-host two-way communication, which underlies several clinical illnesses, such as cardiovascular diseases, neurological disorders, ulcerative colitis, type 2 diabetes, obesity, and cancers [105,106]. Studying gut microbiota changes in aging has attracted increasing attention because of advancements in this field. Similar to zebrafish [37,107-111], one of the areas where telomere shortening occurs most rapidly in humans is the digestive system [112]. The presence of gastrointestinal disorders is frequently linked to severe TBDs [38,113]. The intestinal epithelium of patients with inflammatory bowel disease showed enhanced shortening of telomeres [114,115]. Telomere integrity is thus crucial for maintaining gut homeostasis. In TERT -/- zebrafish, the expression of gut-specific telomerase is capable of preventing gut aging [116]. The health of the entire organism is improved by preventing gastrointestinal tract aging. In TERT -/- zebrafish, reversing intestinal microbial dysbiosis and aging phenotypes has been shown to be adequate to prevent DNA damage, increased p53 expression levels, and cell senescence in this organ [116]. Overall, lifetime extension, while enhancing normal aging, is the most important systemic consequence of gut-specific telomerase activation [116]. As a result, gut telomere-dependent aging regulates organismal aging.

### Cellular senescence

Senescent cells tend to accumulate in aging tissues and contribute to the aging process and related pathologies via a variety of mechanisms. Two of the most extensively researched mechanisms include limiting the replication capacities of stromal cells, immune cells (commonly referred to as immunosenescence), and tissue stem cells (as is often observed in the context of stem cell exhaustion, which is another hallmark of aging); the second mechanism involves disruption of organ function as a result of the release of several proinflammatory molecules, including but not limited to, interleukin-6 (IL-6) and tumor necrosis factor-alpha (TNF-α) [16,117-119]. For the latter, telomere damage/shortening activates the p53/p19ARF and p16Ink4a/Rb signaling pathways, leading to cell senescence and proliferation arrest [120,121]. Additional research is necessary to determine whether and how telomere dysfunction initiates the senescence pathway in stromal tissues with a modest rate of proliferation in normal aging. However, it is intriguing to hypothesize that telomere damage caused by reactive oxygen species (ROS) results in the production of TIF and the initiation of senescence.

### Exhaustion of stem cells

An essential aspect of aging is the depletion of tissue stem cells. p53-dependent apoptosis eliminates tissue stem cells in late-generation TERT- or TERC-null mice as a result of increasing telomere erosion, which contributes to organ atrophy, especially in highly proliferative tissues with high rates of self-renewal, such as the skin, intestinal tract, testis, liver during regeneration, and blood [54,55,57,116,122-131]. TERT plays nonclassical roles that involve activation of the WNT pathway, a key modulator of stem cell homeostasis, which may influence stem cell biology in addition to telomere preservation. In particular, TERT may directly interact with BRG1 in cells from mice and function as a cofactor in the β-catenin complex, thereby upregulating expression of the genes in the WNT network [132].

### Altered cell-to-cell crosstalk

Aging is associated with gradual changes in intercellular crosstalk that increase system noise and impair hormetic and homeostatic modulation. Consequently, aging entails impairments in neuronal, neuroendocrine, and hormonal signaling pathways, such as the dopaminergic, adrenergic, insulin/IGF1-based, and renin-angiotensin systems, along with sex hormones corresponding to the decline of reproductive activities [133,134]. The culmination of these abnormalities in intercellular crosstalk is a characteristic of aging in itself. According to previous data, the fate of T-cells concerning aging is quickly decided after the first synaptic connections with APCs, awaiting telomere transfer [89] [95]. The process of intercellular transfer of telomeres represents an alternative mode of decentralized immunity [101]. As described, the transfer of telomeres from APCs serves to promote the generation of long-lived memory T-cells, circumventing senescence. Decentralization suggests that the extension of telomeres by T-cells is not solely dependent on telomerase molecules [89].

### Genomic instability

Another hallmark of aging is genomic instability, which can cause inflammation and deplete stem cells. Cytogenetic investigations of late-generation TERC-null tissues and cells have demonstrated that telomere malfunction could promote chromosome instability. The results of these investigations have also revealed the formation of anaphase bridges, translocations, deletions, and amplifications in malignancies, such as colorectal cancers (detailed below) [55,122,135-137]. Mechanistically, telomeres that have undergone erosion or uncapping are prone to end-to-end fusion, leading to the formation of dicentric chromosomes, which ultimately gives rise to breakage-fusion-bridge (BFB) cycles. These cycles contribute to chromosomal abnormalities, such as aneuploidy, tetraploidization, translocations, and amplifications, which in turn cause genomic instability via chromothripsis (chromosomal reconfigurations in clusters) and kataegis (localized hypermutations) [59,135,136,138-141]. Additionally, the depletion of p53 makes it possible for cells to survive DNA double-strand-breaking episodes, leading to abnormal chromosomal imbalances as well as nonreciprocal translocations that contribute to carcinogenesis (see “TELOMERES AND TELOMERASE IN AGE-RELATED DISEASES AND CANCER”). Such chromosomal alterations have been observed in aged nonmalignant stem cell microenvironments. The accumulation of mutations in these regions aligns closely with advancing age and has been documented across various human tissue types, such as colonic crypts and the hematopoietic system [142,143].

### Mitochondrial dysfunction

Intimate connections exist between mitochondria and telomeres [126,131]. Age-related reductions in mitochondrial activity result in decreased ATP synthesis and elevated intracellular ROS. Reduced energy generation results in general weakness, whereas a surge in ROS damages cells and forms DNA lesions with the 8-oxoguanine base (telomeres have a high concentration of guanine.). Results of studies using mice with mutations in the mitochondrial polymerase POLG have provided evidence that mitochondrial degradation plays a role in triggering the aging process. Mitochondria with aberrant structures are abundantly reduced (e.g., exterior membrane disruption and cristae fragmentation) [144] and exhibit signs of premature aging in organisms (e.g., cardiomyopathy, decrease in subcutaneous fat, osteoporosis-related decrease in bone mineral density, anemia, decreased body weight, kyphosis, and alopecia). Furthermore, the characteristics of premature aging linked to mitochondrial dysfunction are associated with those seen in telomerase-deficient mice, p53- hyperactivated mice [145], and PGC1α/β-null mice [146]. In light of the critical role played by mitochondria in the aging process, the shared symptoms observed in PGC1α/β-, TERC-, and POLG-null mice have allowed researchers to establish a connection between telomeres, oxidative defense mechanisms, and mitochondria in the context of aging. Notably, there is evidence of decreased oxidative defense and compromised mitochondrial activity in TERC-null mice [126,131]. Additionally, transcriptome investigation of diverse tissues from late-generation TERC-/- animals showed the considerable expression of target genes for p53 and PGC1α/β, thereby allowing researchers to uncover a link between the three opposing hypotheses of aging, a buildup of genotoxic stress, deteriorating mitochondrial function, and rising oxidative damage, by incorporating them. PGC1α/β expression is specifically repressed by p53, which is activated by telomere dysfunction [126,131]. Reduced PGC1α/β expression, in turn, causes a decline in mitochondrial biogenesis and functionality as well as a reduction in the expression of genes involved in oxidative defense. Elevated ROS generation is caused by the signaling circuit of telomeres→p53→PGC1α/β→mitochondria, which also causes 8-hydroxydeoxyguanosine-induced alteration of guanosine bases at telomeres. This axis links telomere dysfunction, mitochondria, and oxidative stress pathways, causing a feed-forward cycle that accelerates aging.

### Epigenetic dysregulation

Some of the age-dependent alterations in the epigenetic environment encompass increasing local methylation and declining global methylation, increasing H4K20 trimethylation, increasing H3K4 trimethylation, increasing H4K16 acetylation, and decreasing H3K9 monomethylation and H3K27 trimethylation [147]. The modulation of sirtuins (SIRTs), a family of seven (SIRT1-7) NAD+-dependent deacetylases that regulate life and health span, reflects explicit telomere and epigenetic interactions [148]. SIRT1 modulates key biochemical processes linked to stress resistance, metabolic activity, and oxidative defense in mammals by deacetylating many aging-relevant transcription factors, such as NF-κB, PGC1α, FOXO, and p53 [149-154]. The p53-mediated suppression of all seven SIRTs caused by telomere dysfunction provides evidence for the interaction between telomere dysfunction and SIRTs (including SIRT1 and SIRT6) [155]. Furthermore, p53 activation caused by telomere dysfunction inhibits PGC1α/β expression, which in turn controls the production of mitochondrial SIRTs 3, 4, and 5. Additionally, the stimulation of p53 causes the transcription levels of three microRNAs (miR-145-5p, miR-26a-5p, and miR-34a-5p) to be upregulated, which inhibits the translation of nonmitochondrial SIRTs 1, 2, 6, and 7 [155]. As a result, telomere-regulated epigenetic pathways are important for regulating the aging process.

### Proteostasis loss

Another possible factor in the aging hallmark of impaired proteostasis is telomere dysfunction as well as the subsequent p53-elicited suppression of SIRT1 expression. A decline in the activities of the chaperone network, which controls maintenance of normal protein-folding capability, leads to proteostasis loss. Rapid aging characteristics have been observed in mutant mice that lack the carboxy terminus of the heat-shock family cochaperone HSP70-interacting protein (CHIP). Heat-shock genes, such as HSP70, are more effectively activated through transcription in mammalian cells when HSF-1 is deacetylated by SIRT1. Therefore, it is conceivable that protein homeostatic mechanisms and heat shock reactions are impaired by telomere dysfunction-elicited suppression of SIRT1 and a resulting decline in HSP70 levels under stress [156]. Protein folding is crucial for preserving the homeostatic balance of neurons. Postmitotic long-lived neurons are particularly sensitive to misfolded proteins because they are unable to remove misfolded proteins via cellular division [157-159]. Several aging-related neurological disorders, including Parkinson's disease (PD) and Alzheimer's disease (AD), are characterized by deregulated protein folding of α-synuclein and β-amyloid peptides, respectively. Cognitive decline and progressive neuronal death are the results of accumulation. Recent research has demonstrated the neuroprotective effect of overexpressing HSP70 in rat, mouse, and Drosophila models of PD and AD [160-164].

### Disabled macroautophagy

The process of sequestering cytoplasmic contents in two-membrane vesicles called autophagosomes, which subsequently merge with lysosomes to destroy luminal material, is known as macroautophagy/autophagy [165]. Consequently, in addition to being engaged in proteostasis, autophagy impacts nonproteinaceous macromolecules (e.g., lipid vesicles, glycogen, and ectopic cytosolic DNA) and entire organelles (e.g., damaged mitochondria that are the target of "mitophagy," as well as other organelles that trigger "pexophagy," "reticulophagy," or "lysophagy"), and invasive pathogens (‘‘xenophagy’’) [165]. Among the most significant causes of decreased organelle turnover is an age-associated reduction in autophagy, which justifies its consideration as a novel sign of aging. According to reports, several metabolic enzymes move into the nucleus and control the silencing of telomeres [166-168]. Serine-responsive SAM-containing metabolic enzyme (SESAME) promotes silent information regulator (SIR) complex binding at telomeres and prevents autophagy-elicited SIR2 disintegration by phosphorylating H3T11 at telomere domains to promote the silencing of telomeres [169]. It has also been demonstrated that autophagy interferes with telomere silencing by destroying proteins linked to telomeres [170]. Through the inhibition of Dot1-catalyzed H3K79me3, the phosphorylation of histone H3T11 (H3pT11) has been shown to reduce the expression of autophagy-related genes and thereby impede the autophagic pathway. Additionally, H3K79me3 and H3pT11 interact to improve the binding of the SIR complex to telomeres to sustain the silencing of telomeres [170]. Moreover, Reb1 recruits SESAME for the phosphorylation of H3T11 at telomeres and limits H3K79me3 at the heterochromatin-euchromatin border to maintain telomere silencing [170].

**Figure 5.**
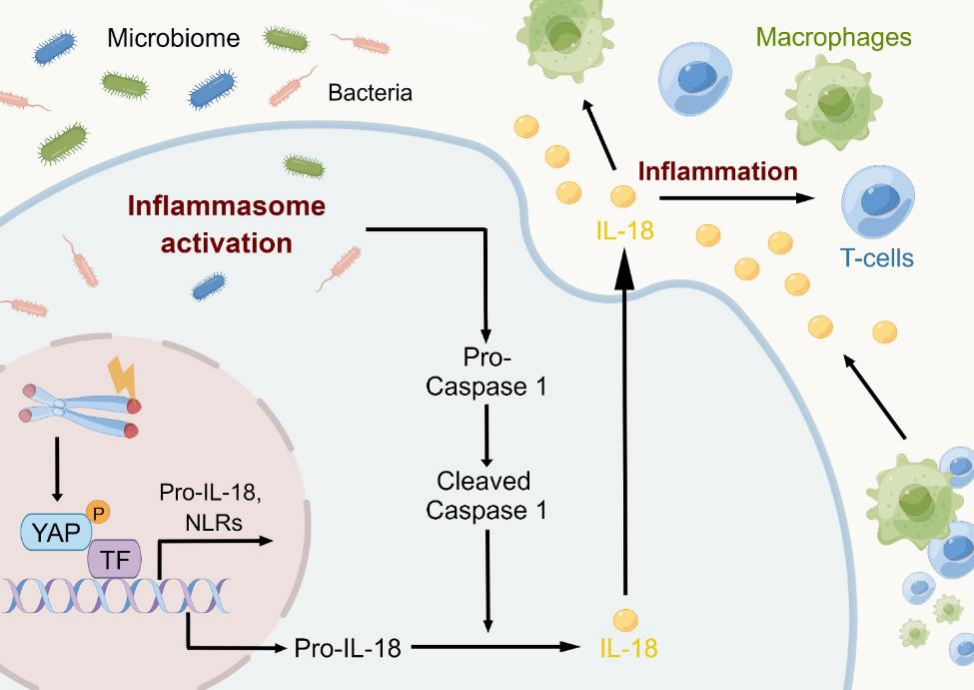
**Tissue inflammation is caused by dysfunctional telomeres**. By activating the ATM/cABL/YAP1 axis and causing the release of mature IL18 to draw in and activate T cells and macrophages, dysfunctional telomeres cause tissue inflammatory reactions. IL 18, interleukin 18; YAP1, Yes-associated protein1; cABL, Abelson murine leukemia viral oncogene homolog 1; ATM, Ataxia telangiectasia mutated.

### Altered Nutrition Sensing

The mTOR-S6 and IGF-1 signaling pathways, as well as proteins from the FOXO and AMPK families, compose an extremely highly conserved network that is involved in deregulated nutrition sensing in aging. To keep the metabolic system in balance, these pathways reciprocally control one another. A key nutritional sensor called AMPK controls mTOR signaling and activates SIRT1 and the FOXO transcription factors transcriptionally [171]. FOXO and PGC1α expression are necessary for the biogenesis of mitochondria and can in turn be activated by SIRT1 [172,173]. As a result, telomere dysfunction can affect metabolic activity since it activates p53 and suppresses PGC1α and SIRT1 activity. Moreover, abnormalities in gluconeogenesis controlled by p53-driven suppression of PGC1α/β, together with the corresponding downstream effectors GLC-6-P and PEPCK, cause dysfunctional telomeres in mice and render them unable to maintain stable plasma glucose levels after fasting [126]. PGC1α/β, PEPCK, and GLC-6-P levels are upregulated, and gluconeogenesis is restored, by forced expression of PGC1α or mTERT or by genetically eliminating p53 [126]. The dependence of tissues on glucose metabolism increases as a result of mitochondrial impairment caused by telomere dysfunction [174]. Alterations in NAD/NADH pools could result from improvements in glycolysis versus mitochondrial oxidative metabolism, which would further impair SIRT activity. Furthermore, telomere dysfunction promotes DNA damage signaling and PARP stimulation, which lowers NAD pools and limits SIRT activity. SIRT function is boosted in brown adipose tissues and muscles when PARP is suppressed or genetically ablated, as was seen in PARP1 knockout mice. PARP and SIRTs compete for the same pool of NAD+ [175].

**Figure 6.**
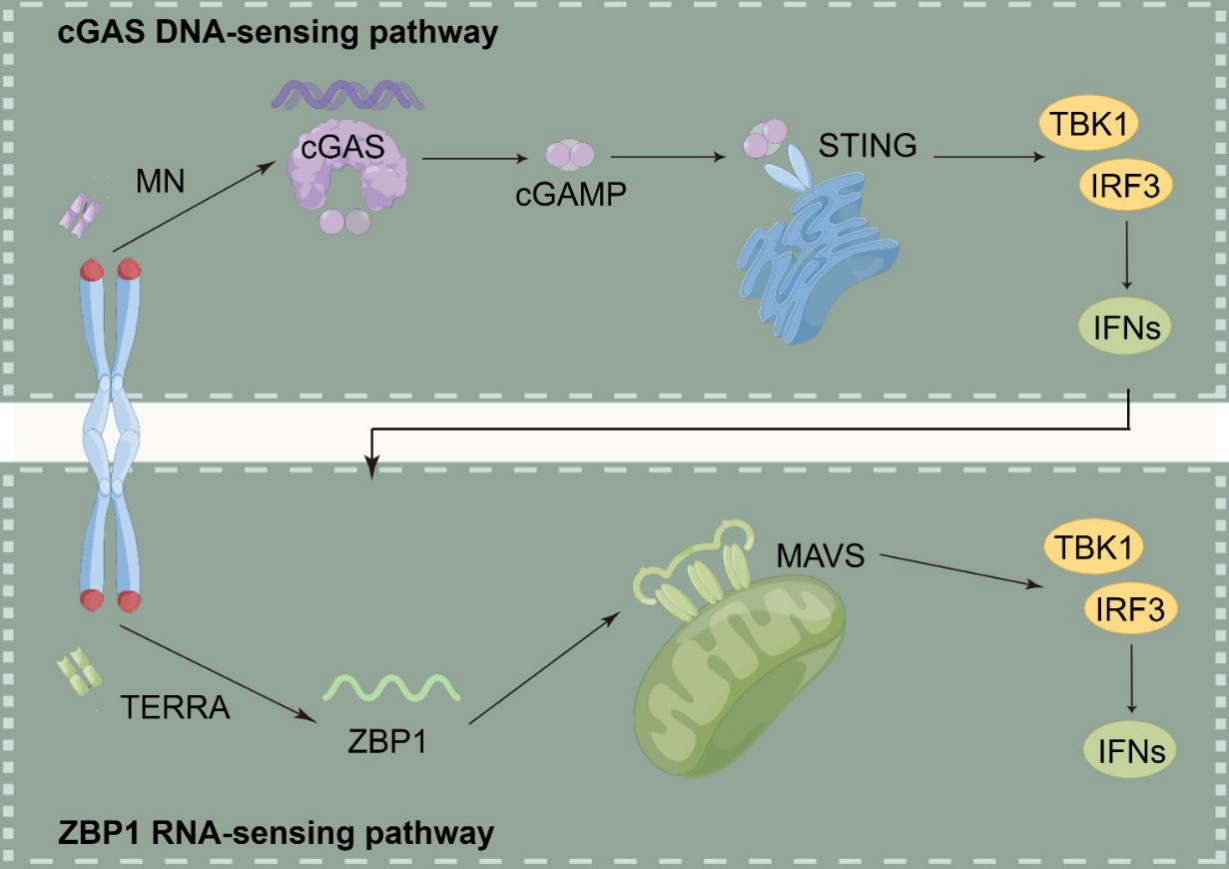
**Two different yet functionally interrelated innate immune sensing pathways must be activated concurrently to promote crisis-related cell death**. The production of various ISGs is primed by cGAS, which also identifies nuclear DNA species delivered to the cytosol as byproducts of breakage-fusion-bridge (BFB) cycles. ZBP1 is activated, and by identifying TERRA molecules derived from unprotected telomeres, it additionally strengthens innate immunity. This causes ZBP1 to self-oligomerize into mitochondrial filaments that can activate MAVS. The removal of abnormal cells containing unstable telomeres is mediated by the continuous synthesis of type I IFNs as well as other inflammatory markers brought on by the stimulation of DNA- and RNA-sensing pathways. Abbreviations: MAVS: mitochondrial antiviral-signalling protein; IFNs: interferons; MN: micronuclei; ZBP1(S): short isoform.

### Inflammation

Inflammatory signals from senescent or genomically defective cells are a component of the aging process. On many different levels, inflammation may be initiated and maintained by telomere dysfunction. First, telomere disruption causes cellular senescence, which increases the synthesis and release of inflammatory mediators such as TNF-α and IL-6 [16,117,119]. Second, the cGAS/STING pathway can be initiated by telomere dysfunction, producing extrachromosomal segments that could hasten autophagy [13]. According to recent research, a novel medication called 6-thio-2'-deoxyguanosine (6-thio-dG) that targets telomeres in cancerous cells causes the production of TIFs and the discharge of extrachromosomal DNA into the tumor microenvironment (TME). Dendritic cells take up this DNA, which stimulates the intracellular cGAS/STING pathway and initiates an inflammatory cascade by upregulating Type I interferons (IFN-Is) through the IRF3/TBK1 pathway. This enhances cross-priming activities and attracts IFNγ-synthesizing CD8+ T lymphocytes [176]. The latest evidence has established a connection between tissue inflammatory responses and telomere dysfunction (Fig. 5) [177]. In particular, telomere dysfunction promotes YAP1 phosphorylation by ATM/cABL, which leads to its nuclear translocation. NLRP6, NLRP1b, NLRC5, and pro-IL-18 are among the inflammasome- and inflammation-related genes that are upregulated by nuclear YAP1. Activation of the inflammasome pathway by the gut microbiota, in conjunction with other factors, leads to the activation of caspase-1. This cleaves pro-IL-18, resulting in the formation of its mature IL-18 form. The secretion of mature IL-18 causes recruitment and activation of T-cells, ultimately initiating an inflammatory response. The in vivo administration of antibiotics, YAP1 inhibitors, caspase-1 inhibitors, or telomerase activators to mice with dysfunctional telomeres reduces the synthesis of mature IL-18 and the cleavage of procaspase-1 to caspase-1, decreasing tissue inflammatory responses as a result. Prior evidence [177] has revealed a direct connection between telomere dysfunction and inflammatory processes, which clarifies in part why inflammatory responses increase with age in elderly people.

This research also demonstrates how the microbiota and cell-intrinsic molecular pathways work together to trigger an inflammatory reaction. In summary, multiple studies have linked telomere dynamics to the molecular mechanisms that underlie all aging-related symptoms. Furthermore, several of these stress responses result in feedback loops that worsen telomere dysfunction, amplifying and hastening the phenotypes of aging. The aging process is both initiated and accelerated by the interconnection of telomeres with essentially all aging characteristics.

**Figure 7.**
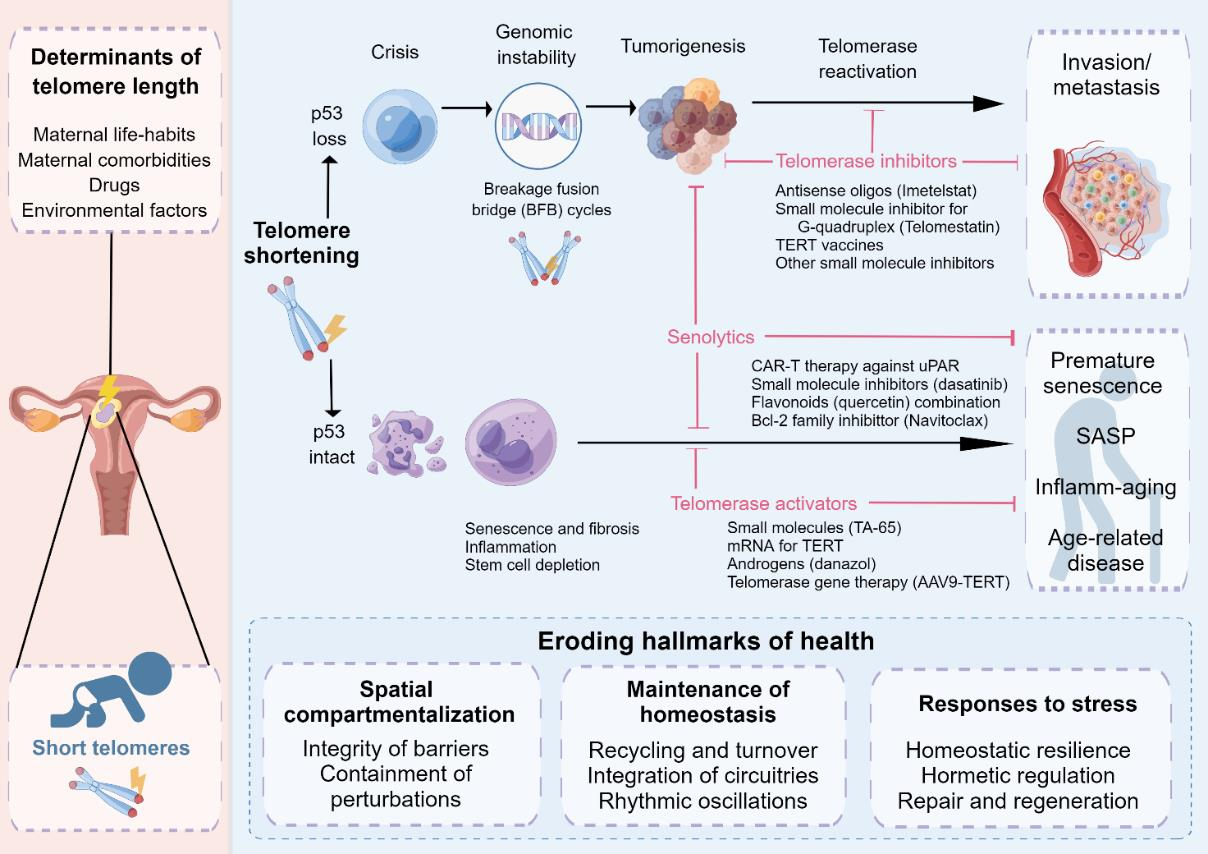
**Telomeres and telomerase in malignancy, aging, prospective treatments, and health**. By modifying the telomere/telomerase axis in embryonic cells, maternal lifestyle choices, medications, and environmental variables may influence the length of telomeres in neonates. When the p53 checkpoint is active, telomere reduction in length causes senescence, fibrosis, apoptosis, inflammatory responses, and depletion of stem cells. Aging and many inflammatory and degenerative disorders are caused by these mechanisms. These mechanisms, as well as aging and disorders associated with aging, can be inhibited by telomerase activators and senolytics. Telomere fusion, as well as break-fusion-bridge cycles, are also brought on by short telomeresThese occurrences result in carcinogenesis when a p53 checkpoint is absent. The advancement to invasion and metastatic spread is brought on by subsequent telomerase activation. Senolytics and telomerase antagonists block the development, invasion, and metastatic spread of tumors. Lastly, there is a connection between the eight markers of health and telomere erosion. SASP, senescence-associated secretory phenotype.

## THE UTERINE CONDITION CAN AFFECT TELOMERE LENGTH IN NEWBORNS

Recently, Muñoz-Lorente et al. asserted that growing embryonic stem cells (ESCs) in the right medium could also result in telomere lengthening. A comparable outcome was also attained for human embryonic stem (ES) cells [178]. These findings support the need for more research into the microenvironmental landscape influencing telomerase expression and/or function in the early weeks of pregnancy instead of implying that human life may be extended by manipulating the telomerase pathway. The telomere/telomerase axis in ES cells may be hindered (or enhanced?) by maternal lifestyle choices, medications, and environmental pollutants. This hypothesis is highly supported by the finding that telomere dysfunction occurred in the neonates of women who were subjected to unhealthy levels of air pollutants [179]. In particular, second-trimester air pollution exposure was adversely related to cord blood characteristics and telomere length in the placenta, thereby identifying an important sensitivity exposure window [179].

It is conceivable that the exchange of information contained in extracellular vesicles may moderate the impact of environmental factors within the uterus. In this approach, extracellular nanovesicles purified from the plasma of young mice could lengthen their lifespan [180,181]. Furthermore, recipient cells exhibit anti-inflammatory traits after being exposed to nanovesicles derived from centenarian cells [182]. Remarkably, extracellular vesicles from young mice exhibited pro-health impacts on aged animals that were associated with those shown in mice having hyperlong telomeres [181,183].

The possibility that environmental practices may influence telomere homeostatic balance during prenatal development and, consequently, have a significant impact on the length of telomeres at which telomere degradation begins is worth studying. A summary of this problem is presented in Figure 7. According to this theory, we may hypothesize that young extracellular vesicles, which can influence telomeric homeostasis, might be present in a young milieu. Evidence in favor of the concept that blood-associated variables may help prolong life in animal models [184] formed the foundation for the very first human clinical study to use plasma from young, healthy donors to treat AD patients [185]. All things considered, a new era in research focused on delaying the negative impacts of aging is imminent.

## CORRELATION OF AGE-RELATED DISEASES AND CANCER WITH TELOMERES AND TELOMERASE

Research on mice and humans with germline mutations in telomere maintenance genes has revealed the role of telomeres in aging and age-associated disorders. Telomere regulation and length vary between murine and human cells. Telomeres in the laboratory mouse (Mus musculus) can reach up to 10 times longer than those in humans (30-150 kb for mice as opposed to 10-15 kb for humans) [44]. Additionally, compared to the modest activity reported in somatic cells in humans, those in mice have higher levels of telomerase activity [186,187]. The engineering as well as characterization of mice having shorter, more human-like telomere lengths has allowed for the clarification of the role of telomeres together with telomerase in genomic stability, aging, and malignancies as a result of these species variations. In particular, consecutive intergenerational crossings in TERT- or TERC-deficient mice progressively shorten telomeres, resulting in telomere dysfunction (end-to-end fusions) by generation G3 [122,135]. This telomere dysfunction is associated with extensive loss of tissue stem cells, gradual tissue atrophy, depletion of germ cells, decreased fertility, deficient adaptive immunity, impaired memory, hindered healing of wounds, reduced stress reactions, increased graying of the hair and alopecia, reduced cardiac performance, weakened skeletal structure, higher incidence of cancer, and general frailty [55, 57, 123, 124, 126, 135, 188-190].

This section outlines the growing body of research connecting telomere function to the aging process, including frailty and age-related illnesses such as neurological disease (PD), metabolic diseases (type II diabetes), cardiovascular diseases (coronary artery disease, vascular dementia, atherosclerosis), and cancers.

### Age-related disorders associated with telomere dysfunction and telomerase

Telomere dysfunction has been linked by several studies to age-associated disorders. First, chronic tissue renewal and decreased levels of telomerase in progenitor cellular compartments in highly proliferative tissues, such as the epidermis, intestinal system, and hematopoietic system, result in gradual telomere attrition over time. Relevant literature, which showed spermidine-mediated age protection was associated with decreased telomere attrition should be included [191]. The result of this attrition is DNA damage, which leads to reactions, such as cell cycle arrest, apoptotic activities, poor differentiation, and/or senescence. Second, ROS-elicited telomere sequence degradation might eventually lead to attrition and uncapping in low-proliferative organs, including the liver, brain, and heart. In particular, research on mice and humans has demonstrated that oxidative stress directly hastens the loss of telomeres in vascular endothelium [192], skeletal muscles [193], cardiac myocytes [194], and adaptive as well as innate immune cells [195,196]. Additionally, TIFs can be produced by ROS-induced guanine alteration, suggesting uncapping from shelterin disengagement, in addition to telomere shortening per se [197]. This is supported by groundbreaking research from the de Lange laboratory that shows how alterations to components of shelterin, including dominant-negative mutant variants of TRF2, may generate TIFs without affecting telomere length [50].

The interplay between senescence and inflammatory processes may be especially pertinent to the telomere-aging relationship. These pleiotropic effects may contribute to the development of AD, osteoarthritis, type II diabetes, PD, and atherosclerosis. In particular, a recent report emphasized the contribution of senescent cells to the development of AD [198]. In this work, microglia and senescent astrocytes that express p16INK4a, which cause tangles in the neurous and Alzheimer's-like phenotypes in mice with mutant Tau (MAPTP301SPS19), were eliminated genetically or pharmacologically, thereby preserving cognitive functions. [198]. This study's relevance arises from the observation that the development of neurofibrillary tangles is preceded by the accumulation of senescent cells, implying that senescent cells might determine how tangles form. Likewise, removing senescent cells extends the lifespan of the BubR1 progeroid murine model [199]. These results initiated the creation of "senolytic" drugs that may eliminate senescent cells in humans [200]. Patients suffering from end-stage coronary artery disease, cardiac hypertrophy, and heart failure could also exhibit the characteristics of an active senescence-inflammation axis [201].

### Inflammatory diseases associated with telomere dysfunction

Research on telomeropathy has shown that telomere dysfunction could cause inflammatory illnesses in aging humans (Fig. 2). Because telomere shortening and tissue inflammatory responses are thought to be caused by elevated ROS generation, telomere shortness and damage in elderly adults without the traditional germline mutations that influence telomeres may contribute to several inflammatory disorders, even when hereditary abnormalities in telomere maintenance genes are absent [202]. These disorders might be caused by some cells in the afflicted tissue having defective telomeres, which causes a locally elevated release of inflammatory factors, additional tissue damage, and telomere shortness. These inflammatory conditions include inflammatory bowel disease [115,203], pancreatitis [204], nonalcoholic fatty liver disease [205], chronic obstructive pulmonary disease [206], and liver cirrhosis caused by chronic liver disease [202]. Dysfunctional telomeres could act as cofactors in these circumstances, amplifying the main causes of the illness.

Senescence and inflammatory processes are especially connected to mitochondrial dysfunction as well as the concomitant rise in ROS production. Since PGC1α upregulation has been demonstrated to have positive impacts on a variety of biological activities that are important to aging in mice and humans, particularly metabolism and ROS modulation, maintaining mitochondrial function is a top treatment goal [207]. Additionally, it has been recognized that unchecked accumulation of resulting ROS damage has an effect on β-oxidation, fatty acid metabolism, and gluconeogenesis, which could also lead to metabolic disorders associated with aging, particularly cancers (see “cancer, telomerase, and dysfunctional telomeres”), sarcopenia, inflammatory disorders, diabetes, general frailty, and neurodegenerative disorders, such as amyotrophic lateral sclerosis, PD, AD, and multiple sclerosis [208,209].

### Association of telomerase and dysfunctional telomeres with cancers

One in two men and one in three women in the US are diagnosed with cancers by the time they are 80 years old, rendering it an age-related disorder (National Cancer Institute). Additionally, epithelial malignancies are the most frequently diagnosed types among older individuals. Tumors frequently have multiple structural changes to chromosomes and are aneuploid. Conversely, lymphoma and sarcoma account for the majority of the cancer spectra in aging mice, with epithelial malignancies being far less common [210]. Additionally, there are seldom any chromosomal structural abnormalities seen in these cancers in mice. The foundation of the genetic instability in these tumors and a fundamental mechanism for the prevalence of malignant epithelial tumors in older adults have been revealed by these cross-species discrepancies. In particular, dysfunctional telomeres [135], in the context of p53 loss, allow cells in crisis to withstand chromosomal breakage episodes, thus driving cancer-related gene translocation, deletion, and amplification by designing shorter telomeres resembling those of humans in telomerase-deficient mice (Fig. 7). Notably, along with increased cancer occurrence, these TERC/p53-null mice also exhibit a humanized tumor range with intricate karyotypes. Together with later genomic evidence of dysfunctional telomeres in early-stage human malignancies, these mouse genetic studies [137,211,212] have demonstrated that telomere dysfunction is a significant factor in the development of epithelial cancers in older individuals. In other words, mice are protected from epithelial malignancies because they lack this mutational pathway (promiscuous telomerase expression and extended telomeres).

The complex involvement of telomeres in tumorigenesis was shown by these mouse models, particularly how dysfunctional telomeres promote tumorigenesis while preventing complete malignant transformation [123,137]. For instance, telomere disruption in the Apcmin+/- TERC-null mouse model first enhances the prevalence of adenomas but eventually prevents the development of macroadenomas and improves survival [137]. In potentially cancerous cells undergoing telomere dysfunction, wild-type p53 is stimulated compared to mutated/nonfunctional p53 as a tumor suppressor. One of the most prevalent occurrences in human epithelial malignancies is p53 tumor inhibitor inactivation [213,214], and depending on how p53 functions, telomere disruption may either promote or prevent the formation of cancers. More precisely, increased rates of cancer occurrence and lower survival rates have been observed in late-generation TERC-/- mice lacking the p53 gene [123,135]. However, Ink4a/Arf-null late-generation TERC-/- mice exhibit a considerable decrease in tumor occurrence rates and longer survival [215]. Remarkably, despite p19ARF deficiency, which activates p53 during carcinogenic activation, Ink4a/Arf-null mice nevertheless have a functional response to DNA damage mediated by p53 (e.g., apoptosis) [216]. As a result, the effectiveness of p53-elicited DNA damage signaling and related cellular checkpoint responses determines the significance of telomeres in tumorigenesis.

According to recent research based on single-cell DNA sequencing of human malignancies, numerous tumors develop as a result of a single punctuated genomic episode [217,218] when all clones are dominated by copy number modifications and mutation bursts. Although the mechanisms accountable for punctuated evolution or "episodic instability" in cancer genomes are presently under thorough investigation, evidence from humans and mice strongly suggests that telomere-based crisis and subsequent telomerase reactivation play critical roles in molding tumor genomes and promoting epithelial tumorigenesis, particularly in older individuals. Although the human genetic data [219] on developing malignancies are compatible with this episodic instability hypothesis, the two hypotheses are not mutually exclusive, and the “Vogelstein mechanism” [220]—the gradual aggregation of driving mutations—may also be at play.

Further corroboration substantiating the association between telomeres and cancer entails the age-related degeneration of telomeres in the colonic mucosa of older individuals and the relatively truncated telomeric length found in malignancies compared to neighboring noncancerous tissues [221]. Furthermore, relative to matching healthy tissues, human tumor tissues express TERT and TERC at higher levels [222], and TERT expression must be forced for human primary cultured cells to undergo malignant transformation, showing that complete malignant transformation requires telomere preservation [223]. Last, a telomere-based crisis is initially experienced by cancer-prone animals bred with an inducible TERT allele, after which telomerase is reactivated. This crisis-reactivation sequence results in extremely aggressive tumors, suggesting that a crisis makes it possible to acquire genome events that promote the aggressiveness of a malignant phenotype [211,224]. Similarly, transient induction of telomere uncapping in telomerase wild-type animals (by disrupting the shelterin complex and mutant TRF2 protein) causes an increase in the development of hepatocellular carcinoma (HCC) in mice exposed to carcinogens [225]. In contrast, in a model of HCC established by carcinogen exposure, the shortening of telomeres in TERC-knockout mice enhances tumorigenesis but does not cause malignant transformation [226]. Hence, a variety of data support the notion that telomere-based crises and telomerase reactivation perform distinct roles in the occurrence and progression of cancers.

### Tumor-suppressive mechanisms dependent on telomeres and telomerase

Previous research has identified a method for telomere-mediated tumor inhibition in which defective telomeres in crisis drive two interconnected cytosolic nucleic acid sensing mechanisms and cause a fatal interferon (IFN) response (Fig. 6). The early stimulation of the cGAS/STING pathway and production of IFN-stimulated genes (ISGs), particularly ZBP1, are triggered by the breaking of fused telomeres and the consequent transfer of nuclear DNA into the cytoplasm. Nevertheless, ZBP1(S) must be further activated downstream by TERRA, and ZBP1 filaments need to assemble on mitochondria for crisis-related cell death to occur. This encourages an inflammatory cycle that causes the production of an ISG profile and the triggering of autophagy via an as-yet-unidentified route, which particularly causes cell death in replicative crises [227].

Precancerous cells with destabilized telomeres could be destroyed by the effective cell death response mediated by IFN-I in the innate immune system when the ZBP1-RNA-sensing and cGAS-DNA-sensing pathways are both simultaneously active [227]. These results reveal an interactive relationship between extremely short telomeres, innate immunity, and mitochondria that has developed to prevent the development of age-related cancer in humans.

### Carcinogenic mechanisms dependent on telomeres and telomerase

End-to-end fusion is initiated, as previously mentioned, by telomere function degradation. These dicentric chromosomes can produce anaphase bridges when undergoing mitosis and the ensuing chromosomal breakage, resulting in the breakage-fusion-bridge (BFB) cycle, as initially suggested by Barbara McClintock [228]. During the crisis, the surviving BFB daughter cells gather chromosomal abnormalities associated with tumors, and they eventually activate telomerase to stifle DNA damage signals and control chromosomal instability, allowing malignant growth. This theory of cancer genomic evolution has been empirically supported in a prostate cancer TERT-inducible model. In contrast to telomere-proficient controls in this cancer model [211], tumors with extremely short telomeres are relatively small and far less aggressive since they exhibit a higher cancer cell death rate and reduced proliferative rate. The reactivation of telomerase experimentally causes the malignancy to progress, such as the acquisition of additional characteristics, such as bone metastasis, in these high-grade prostatic intraepithelial neoplasias with unstable chromosomes. Conversely, controls with perfectly preserved telomeres (which do not go through a crisis accompanied by telomerase reactivation) only display local invasion [211]. Additionally, genome examination of the tumors in the crisis has revealed more prometastatic alterations (for instance, deletion of SMAD4 and further perturbations to the TGF-β pathway). As a result, a telomere-based crisis causes the instability of chromosomes to trigger carcinogenesis, and telomerase reactivation subsequently confers ongoing proliferation capacities to these cells [229].

The most prevalent noncoding variations in human malignancies, according to large-scale sequence analyses, are TERT promoter mutations [230-232]. Such mutations are significantly overrepresented in malignancies developing in tissues with poor rates of self-renewal [233]. For instance, mutations in the TERT promoter are particularly prevalent in melanoma, TERT expression is often increased by a factor of four [234], and > 80% of primary glioblastomas exhibit TERT promoter mutations [233]. The G228A and G250A single-nucleotide mutations are the most frequently recurring alterations in the TERT promoter, which can produce de novo ETS consensus binding domains to bind to GAPB and perhaps other ETS transcription factors, thereby increasing the expression of TERT [235]. Chromosomal changes caused by a crisis are another factor contributing to enhanced TERT expression in malignancies [135], which results in HCC TERT locus amplification [236]. Last, investigations on gene therapy have shown that restoring the TERT promoter to its wild-type sequences using CRISPR greatly increases the longevity of mice with glioma [237].

TERT gene transcription can be improved by carcinogenic signaling pathways in addition to the genetic processes promoting TERT overexpression. Telomerase activity is activated in human primary fibroblasts when c-MYC binds to Myc binding domains in the TERT promoter, even though forced TERT expression is an inadequate replacement for c-MYC in inducing transformation [215,238]. Additionally, active KLF4 and WNT signaling interact to induce TERT transcription [239]. TERT can be activated by specific pathways involved in development and carcinogenesis, but the ability of TERT to activate these pathways in cis has also been demonstrated. The proliferation of dormant renal podocytes and hair follicular stem cells may occur when telomerase is activated in models of mice overexpressing TERT, regardless of TERC activity, telomere length, or TERT reverse transcriptase activity [58,240]. The results of mechanistic investigations have identified that TERT binds specifically to TCF motifs and improves the transcriptional programs of WNT and MYC [132,241], which are mechanisms controlling the stem cell homeostatic balance and promoting cancer growth [242,243]. TERT has also been implicated in interactions with the NF-κB complex and binding to the promoters of TNF-α and IL-6, thus boosting cellular proliferation and preventing cell death [244]. By suppressing p65 expression, the ectopic expression of TERC and TERT attenuates the enhanced cellular proliferative rate and resistance to cell death. In terms of functional resistance to endotoxic shock, G1 TERC-null mice performed better than littermates who had normal TERC expression, with > 50% of mice able to survive lipopolysaccharide-induced shock relative to 25% of controls, implying that telomerase controls NF-κB-induced inflammation without regard to the length of the telomeres. Collectively, these findings show how TERT and carcinogenic signaling molecules interact to control circuits important for cancer metastasis. Further research is needed to determine the significance of these circuits in human cancers.

Shelterin protein mutations and/or altered expression have been found in cancers; however, their exact functions in cancer onset and advancement are yet unknown. Telomere dysfunction in the early stages of chronic lymphocytic leukemia is associated with the development of illness and end-to-end fusions [245]. Consequently, the expression levels of shelterin proteins such as POT1, RAP1, and TRF1 as well as TPP1 and TIN2 are decreased in this illness. Five percent of chronic cases of lymphocytic leukemia had somatic mutations in POT1, the shelterin-associated protein that is most often altered in human malignancies. RAP1 or POT1 somatic mutations may arise in HCC, mantle cell lymphoma, cardiac angiosarcomas, parathyroid adenoma, Li-Fraumeni-like syndrome, familial melanoma, and familial glioma [212]. TRF1 and TRF2 expression are both elevated in a variety of tumor forms, including malignancies of the kidney, stomach, colon, lungs, and breasts, suggesting that shelterin proteins aid in the development of human malignancies.

### Alternative cancer-related telomere maintenance mechanisms

Despite telomerase being recognized as the principal mechanism of telomere maintenance in cancer, tumor cells also use an alternative telomerase lengthening approach, referred to as alternative lengthening of telomeres (ALT), which is recombination-mediated and telomerase-independent [246]. ALT is present in 5-15% of human malignancies, including glioblastoma and osteosarcoma, and is frequently linked to an unfavorable prognosis [247]. By copying telomeric DNA templates from nonhomologous chromosomes or sister chromatids, DNA homology-directed repair processes used by ALT extend telomeres. The following are crucial components for ALT: FANCD2, FEN1, NBS1, RAD50, MUS81, and MRE11 [6,248]. Notably, ALT-positive cells frequently cannot detect cytosolic DNA, and extrachromosomal telomere repeats (ECTRs) have been shown to trigger an interferon (IFN) response by way of the cGAS/STING cytosolic DNA-sensing pathway [249]. Because ECTRs are constantly being produced, cancerous cells using ALT must also overcome additional anti-proliferative obstacles, such as senescence onset and innate immune surveillance. Surprisingly, ALT is less effective at promoting aggressive malignancies and metastasis but is a significant component that promotes carcinogenesis in the absence of telomerase reactivation. Evidence from a research report using passaged mouse mTerc -/- Ink4a/Arf -/- fibroblasts demonstrated the formation of ALT. The fact that telomerase-positive cancerous cells developed the ability to metastasize after mTERC transduction, though ALT-positive cancerous cells did not, shows that ALT-positive and telomerase-positive malignancies are physiologically not similar [250].

For treatments using telomerase inhibitors, the ALT pathway may act as a resistance mechanism. Using a TERT allele under tamoxifen regulation, a model of mice with ATM mutation and susceptibility to lymphoma was created (TERT-ER) [224], and it was found that lymphoma cells once again entered a telomere-based crisis following the cessation of telomerase activity. Two-thirds of the mice were treated by telomerase inhibition, while the other animals had recurring tumors with ALT hallmarks [224]. These recurrent malignancies gained amplifications and deletions in homologous recombination-related genes, thought to be crucial for ALT, and PGC1α locus amplification, which can be an indication of persistent mitochondria stress in ALT-positive cells [224]. The above-described mechanisms provide valuable information for designing possible treatment strategies that involve the use of telomerase inhibitors in combination with therapies that target mitochondria or homologous recombination pathways.

## TELOMERASE IN MEDICINE

Research on telomerase restorative treatment as a possible anti-aging technique has been sparked by the association of telomere dysfunction with the signs and symptoms of aging, the prevalence of age-associated disorders, and the emergence of hereditary and acquired degenerative ailments (Fig. 7). An optimal approach for administering this therapeutic strategy would presumably involve a temporary induction of telomerase to aid in the recovery and rejuvenation of telomere resources while minimizing the risk of promoting cancer through continuous telomerase activation. Notably, mice with increased TERT expression had the lowest percentage of cells with short telomeres; nevertheless, these mice also had more copies of ARF, p16, and p53 [251], which improved their resistance to cancer. Whether the prolonged lifespan and disease-modifying effects of forceful TERT expression are related to the effects of TERT on telomeres or to its stimulation of WNT, which may augment stem cell reserve, is yet unknown.

The search for drugs that increase TERT expression for anti-aging treatments has been sparked by advances in our understanding of the molecular mechanisms governed by telomerase and the regeneration of prematurely aged mice via telomerase activation [124]. It has been discovered that several small molecules seem to stimulate TERT (Fig. 7), such as TA-65 [252] and histone deacetylase inhibitors [253]; however, their exact mechanisms of action are poorly understood. TA-65 (cyclastragenol) is a naturally occurring substance that is extracted from several Astragalus plant types. Recent human TA-65 studies have shown enhanced macular function [254], decreased concentrations of high-density lipoprotein, and decreased levels of the inflammatory indicators CRE and TNF-α [255]. Patients with telomeropathies are also being studied for the effects of hormonal treatments that can raise telomerase levels, including the antiprogestogenic and antiestrogenic drug danazol and the androgen 5α-dihydrotestosterone [256,257]. The Blasco laboratory's proposal of adeno-associated virus (AAV)-mediated delivery of telomerase offers a promising strategy for addressing telomere-related conditions. The use of AAV vectors for gene delivery provides a versatile platform, and preclinical studies have shown encouraging results in terms of telomere maintenance, tissue function, and lifespan extension [258,258]. However, careful considerations regarding targeted delivery, immune responses, precise control of telomerase activity, and long-term safety and efficacy are essential for the successful translation of this approach into clinical practice. Continued research and rigorous evaluation are necessary to further understand and optimize the potential of AAV-mediated telomerase delivery as a therapeutic intervention. Finally, the recent discovery of drugs that target TERC stability could present novel treatment possibilities for telomeropathies. In particular, PAPD5 breaks down TERC via 3' oligoadenylation, preparing these transcripts for RNA exosome degradation. As previously indicated, dyskeratosis congenita is one condition where PARN, a protein implicated in TERC maturation and deadenylation, is altered. Induced pluripotent stem cells from individuals with dyskeratosis congenita had longer telomeres, and animals that received a PAPD5 small molecule inhibitor for a longer duration exhibited high tolerance [259].

Therapy for progeroid disorders such as Werner and Bloom syndromes would be a truly fascinating use of pulsatile telomerase activation therapy. This hypothesis is based on the startling absence of degenerative symptoms in mice deficient in expression of genes linked to hereditary degenerative diseases in humans, such as (i) ataxia-telangiectasia, which is characterized by ATM mutational inactivation; (ii) Bloom syndrome, resulting from a mutation in the RecQ helicase family member BLM; and (iii) Duchenne muscular dystrophy (DMD), resulting from dystrophin, DMD mutation. Surprisingly, each of these genotypes resulted in telomeres that were shorter and more closely resembling those seen in humans when they were crossed with the TERC-knockout mice (typically at G2-G3) [260-263]. For instance, WRN/TERC-null mice replicated the distinctive characteristics of Werner syndrome, such as kyphosis, senile cataracts, graying hair and alopecia, hematopoietic disorders including pancytopenia and an imbalanced immune response, deficiencies in immune and stem cell compartments, pathological long bone fractures, and metabolic disorders, such as insulin resistance [260].

As mentioned, degenerative symptoms are not seen in the telomere-intact murine model exhibiting mutations in the BLM or WRN genes, justifying the use of telomerase activation treatment to prolong life and postpone or minimize the severity of symptoms. Similarly, telomerase stimulation treatment may help patients with germline mutations that impact telomere maintenance (such as DKC) by reversing their advancing symptoms, including gastrointestinal dysfunction, pulmonary fibrosis, and anemia. Chronic inflammatory conditions such as ulcerative colitis, pancreatitis, and liver cirrhosis are another class of disorders with few available treatments in which telomerase stimulation may be helpful (Fig. 7). When a disease first manifests, telomere dysfunction may cause tissue inflammatory responses, which may then speed up telomere shortening, generating a feedback loop that eventually results in disease resurgence and even malignancy due to telomerase reactivation, p53 depletion, and genomic instability. Once more, telomerase activation from the very beginning of the disease before the onset of a telomere-based crisis may be able to stop disease flare-ups and tumorigenesis. The significant increase in indices of mouse brain health upon telomerase genetic stimulation suggests that telomerase activation could be effective in managing neurodegenerative disorders [124,211]. By inhibiting the operation of checkpoints that regulate the tissue-disrupting consequences of dysfunctional telomeres, it may also be capable of reducing telomere dysfunction-induced organ degradation and death in addition to activating telomerase. Furthermore, research has demonstrated that in mice with dysfunctional telomeres, the preservation of tissue integrity and longevity might be prolonged without affecting tumor growth by selectively inhibiting cell cycle arrest mediated by p21 [264] or apoptosis triggered by Puma [265]. Additionally, Exo1 deletion enhances tissue maintenance and longevity in mice with dysfunctional telomeres by preventing the development of chromosomal fusions and BFB cycles, which are responsible for causing DNA damage and activating p53 checkpoints in these mice [266].

The enhanced telomerase activity seen in most malignancies has prompted the establishment of antitelomerase treatments, in contrast to the potential uses of telomerase stimulation in anti-aging intervention. Numerous approaches, such as antisense oligos, vaccinations, and small molecule inhibitors, have been developed to target TERT in malignancies [267-269] (Fig. 7). Nevertheless, no antitelomerase drugs have progressed to randomized phase III studies. It could take some time for telomeres to shrink to a length that might cause tumor reduction, which explains the limited success of this treatment. Alternative approaches to telomerase inhibition may have a more significant clinical impact. First, because functioning checkpoint machinery could cause senescence in tumors with perfectly preserved p53, telomerase suppression would be more effective in these cases. Nonetheless, based on results of preclinical animal research showing that TERT suppression might activate the ALT pathway in lymphoma, this approach must still be used cautiously [224]. Therefore, medicines that block both telomerase and the ALT pathway simultaneously [270] might reduce the development of resistance. The fraction of tumors that are ALT-positive could respond more quickly and have better outcomes if certain immune circuits are targeted. That is, because cancerous cells that are ALT-positive continue to produce cytosolic DNA [249], it is reasonable to hypothesize that these malignancies are more amenable to immunotherapy given that cytoplasmic DNA increases IFN signaling by activating cGAS/STING. According to preclinical research, the nucleoside analog 6-thio-dG could make tumors impervious to anti-PD-L1 therapy and sensitive to immune checkpoint therapy by incorporating the analog into freshly formed telomeres and causing telomeric DNA damage [176]. As a result of its potential function in the suppression of advanced cancers, antitelomerase treatment is still a feasible anticancer method. However, in addition to identifying the precise combination treatments for synergistic efficacy, the biological and genotypic environment will also be crucial.

Last but not least, research into the fundamentals of cell biology and chromosomal organization has elucidated the underlying causes of many serious human disorders. Telomere research has served as an excellent example of fundamental science and interdisciplinary confluence, elucidating the function of telomeres and telomerase in the pathogenic mechanisms that underlie cancers and the characteristics of aging. Various gaps in our knowledge pertaining to telomeres still exist. For instance, we need to investigate and better understand the mechanisms that regulate telomerase expression and activity, as well as the noncanonical roles of TERT. Additionally, we need to gain insight into the interactions between telomere dysfunction and disease-associated processes, such as those involved in degenerative, fibrotic, and inflammatory diseases. Telomere dysfunction undoubtedly performs a crucial pathogenetic function in human diseases, irrespective of whether it causes the illness or is merely a consequence of it. Such a crucial function facilitates the establishment and careful examination of telomerase activators to treat aging and age-related disorders as well as the evaluation of effective telomerase inhibitors for cancer therapy in advanced stages. The characteristics listed herein provide a foundation to inspire more research into the function of telomeres and telomerase, which could aid in addressing aging, the fatal disorder that ultimately affects everyone.

**Table 3:** Examples of anti-aging effects of Telomere attrition-targeted interventions in mammals.

Species/model	Intervention	Outcome	Ref
**mouse**	telomere lengthening	lifespan extension	[251]
**Telomerase null mouse**	telomerase reactivation	lifespan extension	[124]
**mouse**	pharmacological or genetic activation of telomerase	delayed aging	[425]
**mouse**	hyperlong telomeres	increased lifespan; metabolic health improvement	[183]
**mouse**	telomere maintenance in adult neurons	preservation of neuron survival and cognitive function	[426]
mouse	telomerase activation by gene therapy strategy	improvement in models of pulmonary fibrosis and aplastic anemia	[427,428]

## FUTURE PERSPECTIVES

Eight hallmarks of health were recently proposed [104], consisting of the organizational characteristics of spatial compartmentalization (barrier integrity and local disturbance containment), homeostatic preservation over time (rhythmic oscillations, circuitry integration, recycling, and turnover), and a variety of appropriate perturbation responses (resilience of homeostasis, hormetic control, and regeneration and repair). Without a doubt, these eight indicators of health gradually deteriorate as a result of the dysfunction of telomeres, suggesting an increasingly diminished ability to sustain spatial compartmentation (as a result, internal and exterior barriers lose their integrity, and it becomes impossible to restrict changes to these barriers through time and space), to ensure a sustained homeostatic state (diminished potential for recycling and turnover, improper use of integrated circuits for system coordination, and misaligned infradian, circadian, or ultradian rhythms), and to fully repair and regenerate, maintain homeostasis, and regulate hormesis to appropriately respond to stress (Fig. 7). As a consequence, the eight indicators of deteriorating health are linked to dysfunctional telomeres, establishing a multidimensional space of interplay that might clarify certain aspects of the telomere dysfunction mechanism.

In light of the remarkable advancements in longevity strategy development in mammalian model organisms and early clinical trials (Table 3), developing logical approaches to address human telomere dysfunction will be crucial. The issue is whether attempts to prolong human lifespan should be centered around avoiding environmental variables that hasten aging (such as pollution, stress, insufficient exercise, and poor diets, which are frequently inevitable in the context of deprivation, precarity, and conflict), the practice of lifestyle choices that promote health (including healthy eating, exercising, consistent sleep schedules, and social engagements), the use of pleiotropic, generally nonspecific drugs (such as danazol, androgens, navitoclax, quercetin, flavonoids, and dasatinib), or highly targeted medical treatments. Considering the spectrum of hallmarks associated with therapeutic approaches aimed at slowing, stopping, or reversing telomere dysfunction, it would be worthwhile to assess the effectiveness of treatment regimens that involve a combination of interventions designed to optimize therapeutic benefits while minimizing adverse effects. The topic of whether such prophylactic drugs to extend lifespan and health span will benefit from personalization depending on patient features identified by the phenotypic, metabolomic, epigenetic, or genetic evaluations of telomere dysfunction is still unresolved.

At present, telomere dysfunction is not regarded as a formal target for medication development or treatment. Consequently, the initial clinical trials investigating anti-telomere dysfunction therapies must primarily focus on preventing or reducing the incidence of the disorders linked with telomere-related pathologies rather than targeting telomere dysfunction itself. Moreover, noteworthy advancements in telomere research have uncovered novel mechanisms underlying telomere maintenance. For example, one such mechanism involves the regulation of human telomere length through thymidine nucleotides [67], while another mechanism pertains to the transfer of telomeres between cells to rescue T-cells from senescence [89]. These latest discoveries offer a novel viewpoint and suggest new possibilities for the treatment of telomere dysfunction as well as associated illnesses and might offer a better foundation for the development of successful therapies meant to increase healthy lifespan.

## CONCLUSION

In summary, aging research has emerged at the forefront of scientific research due to the growing social and economic costs associated with the growing aging global population. The defining features of aging involve a variety of molecules, and we analyze how telomere dysfunction potentially amplifies or accelerates the molecular and biochemical mechanisms underpinning each feature of aging and contributes to the emergence of age-associated diseases, including cancer and neurodegeneration, via the perspective of telomere biology. Additionally, the recently identified novel mechanistic actions for telomere maintenance offer a fresh viewpoint and approach to the management of telomeres and associated disorders. Telomeres and defining features of aging are intimately related, which has implications for therapeutic and preventative approaches to slow aging and lower the prevalence of age-related disorders.
